# Supplementary material for: In situ electrochemical regeneration of nanogap hotspots for continuously reusable ultrathin SERS sensors
Source: Nat Commun. 2024 Mar 6;15:2022. doi: 10.1038/s41467-024-46097-y (PMC10917746; doi:10.1038/s41467-024-46097-y)
Supplement: Supplementary file 1 — Supplementary Information [file 41467_2024_46097_MOESM1_ESM.pdf]

Supplementary Information

In-situ electrochemical regeneration of nanogap hotspots for continuously reusable ultrathin SERS sensors

Sarah May Sibug-Torres[1], David-Benjamin Grys[1], Gyeongwon Kang[1,2], Marika Niihori[1], Elle Wyatt[1], Nicolas Spiesshofer[1], Ashleigh Ruane[1], Bart de Nijs[1], Jeremy J Baumberg[1*]

[1] NanoPhotonics Centre, Cavendish Laboratory, Department of Physics, JJ Thompson Avenue, University of Cambridge, Cambridge, CB3 0HE, United Kingdom
[2] Present address: Department of Chemistry, Kangwon National University, Chuncheon, 24341, South Korea
* Corresponding author: jjb12@cam.ac.uk

Supplementary Figures

Supplementary Fig 1 | MLagg-CB[5] preparation and initial cleaning with oxygen plasma cleaning (O_2-PC) and solution re-scaffolding. 3

Supplementary Fig 2 | SERS and Raman spectra of MLagg-CB[5] and CB[5] solution.. 4

Supplementary Fig 3 | Analyte removal from MLagg-CB[5] with O_2-PC and solution re-scaffolding. 5

Supplementary Fig 4 | Electrochemical SERS (EC-SERS) flow and optical set-up. 6

Supplementary Fig 5 | Cyclic voltammetry (CV) of MLagg-CB[5] in 50 mM potassium phosphate buffer. 7

Supplementary Fig 6 | Cyclic voltammetry of MLagg-CB[5] in 1 mM CB[5] and buffer.. 10

Supplementary Fig 7 | Linear sweep oxidation and reduction of MLagg-CB[5] in alternating solutions. 12

Supplementary Fig 8 | Potential step oxidation and reduction of MLagg-CB[5] in alternating solutions. 13

Supplementary Fig 9 | Potential-dependent binding of ADN on MLagg-CB[5]. 14

Supplementary Fig 10 | Regional uniformity of ADN signal over different MLagg-CB[5] substrates.. 16

Supplementary Fig 11 | Regional uniformity of ADN signal on MLagg-CB[5] over multiple cycles of analyte detection and cleaning/regeneration with CB[5].. 17

Supplementary Fig 12 | Regional uniformity of ADN signal on MLagg-CB[5] over multiple cycles of analyte detection and cleaning/regeneration without CB[5]. 18

Supplementary Fig 13 | Cycles of 10 µM ADN detection, cleaning and regeneration with buffer and 1 mM KCl on MLagg-CB[5] 19

Supplementary Fig 14 | Cycles of 10 µM ADN detection, cleaning, and regeneration with buffer on MLagg-NaCl.. 20

Supplementary Fig 15 | Cycles of 100 µM ADN detection, cleaning, and regeneration with CB[5] on MLagg-CB[5] with DF measurements at each regeneration step. 21

Supplementary Fig 16 | SERS spectra from blank and spiked urine test samples. 22

Supplementary Fig 17 | Standard additional analysis of ADN in urine. 22

Supplementary Fig 18 | Initial EC cleaning and regeneration of MLagg-CB[5] 23

Supplementary Tables

Supplementary Table 1 | Studies reporting different strategies for SERS substrate recycling or cleaning 24

Supplementary Notes

Supplementary Note 1 | Cyclic voltammetry of MLagg-CB[5] 8
Supplementary Note 2 | Solution switching for oxidation and reduction of MLagg-CB[5]................................11
Supplementary Note 3 | Potential-dependent binding of ADN 15

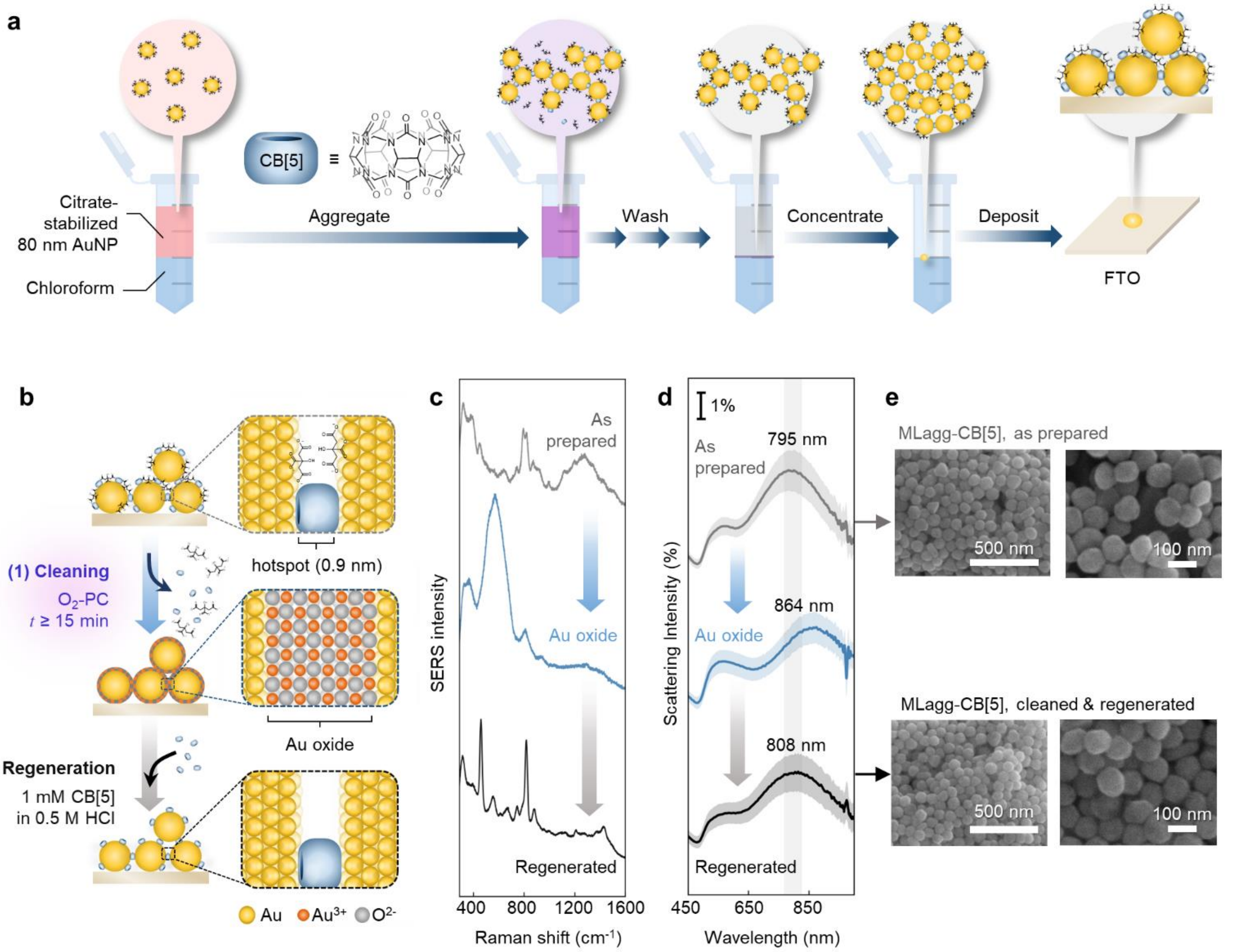

Supplementary Fig 1 | MLagg-CB[5] preparation and initial cleaning with oxygen plasma cleaning (O_2-PC) and solution re-scaffolding. a Step-by-step schematic illustrating preparation of MLagg-CB[5]. **b** Schematic of the initial cleaning and regeneration of freshly prepared MLagg-CB[5] using O_2-PC and nanogap re-scaffolding *via* immersion in CB[5] and HCl. **c** SERS spectra (1 s integration time, 785 nm excitation laser at 1 mW power) and **d** DF scattering spectra from the MLagg-CB[5] as prepared (grey), after O_2-PC (blue), and after CB[5]-regeneration (black). For the DF spectra in **d**, solid lines and shaded area represent mean and ±1 s.d. of n=150 spectra obtained across the area of a MLagg-CB[5]. Grey line highlights initial chain mode peak wavelength. Spectra are offset for clarity. **e** Scanning electron micrographs (SEM) of the MLagg-CB[5] (*top*) as prepared and (*bottom*) after initial cleaning and regeneration.

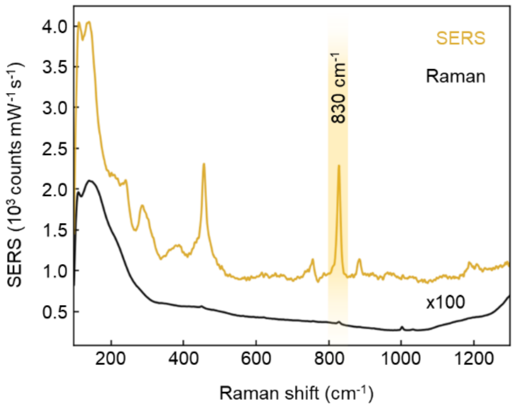

Supplementary Fig 2 | SERS and Raman spectra of MLagg-CB[5] and CB[5] solution. SERS spectrum is measured on a MLagg-CB[5] using 1 s integration time and 2.1 mW 785 nm laser excitation. Raman spectrum of a 2 mM CB[5] solution in water is taken with 200 s integration time and 127 mW 785 nm laser excitation. All measurements taken using a 20x NA 0.40 objective.

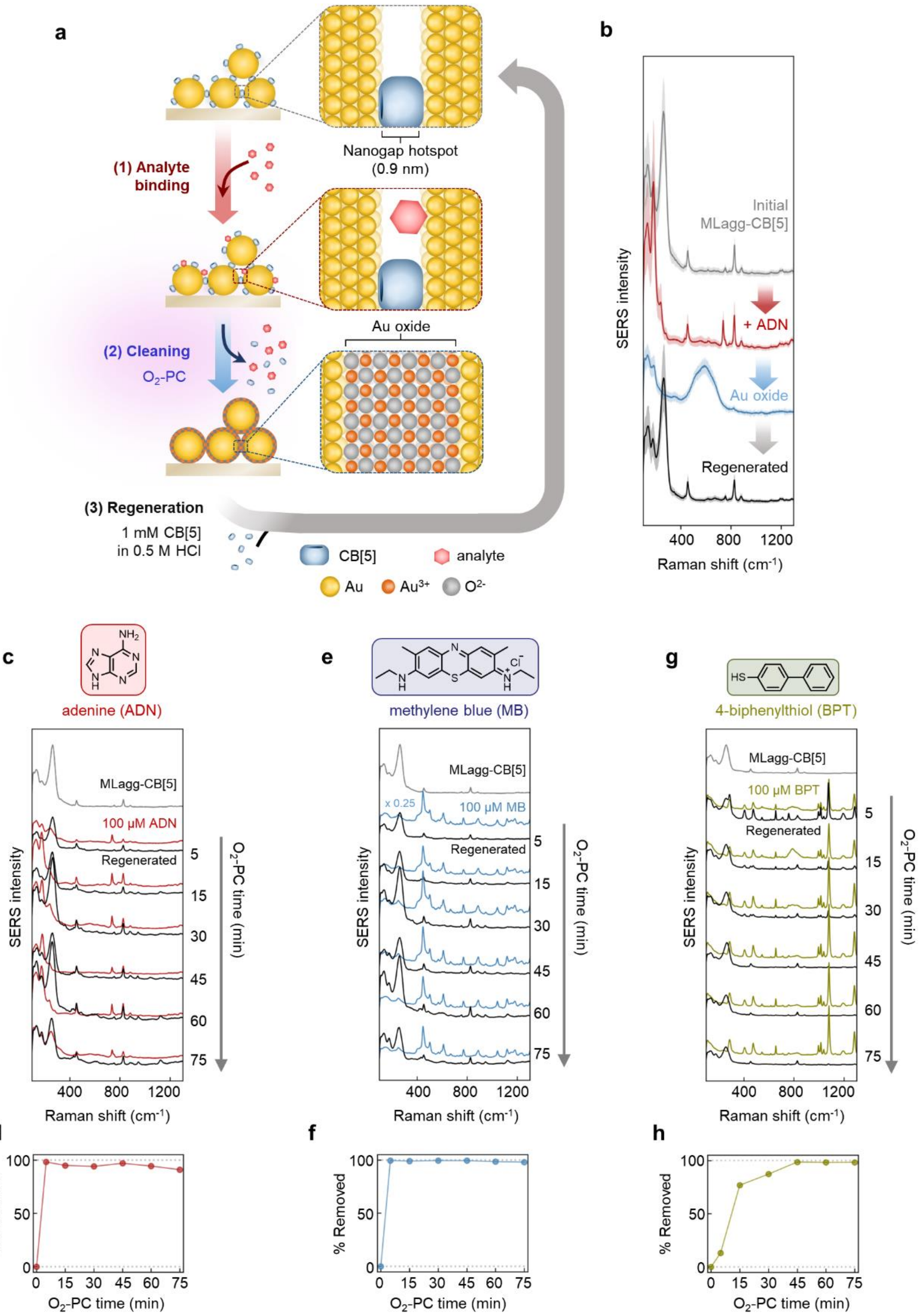

Supplementary Fig 3 | Analyte removal from MLagg-CB[5] with O_2-PC and solution re-scaffolding. a Schematic illustrating the use of oxygen plasma cleaning (O_2-PC) and CB[5] re-scaffolding for cleaning and regenerating the MLagg-CB[5] after analyte detection. **b** SERS spectra from the initial MLagg-CB[5] (grey), after the detection of 100 µM adenine (ADN, red), after 45 min O_2-PC (blue), and after regeneration (black). Solid lines and shaded areas represent the mean and ±1 s.d. of n=10 spectra collected over the MLagg-CB[5] area. SERS spectra from the initial MLagg-CB[5] (grey), after detection of 100 µM of **c** ADN (red), **e** methylene blue (MB, blue), and **g** 4-biphenylthiol (BPT, green), and after cleaning and regeneration at different O_2-PC times (black). Spectra are offset for clarity. % change in **d** ADN, **f** MB, and **h** BPT signal after different O_2-PC cleaning times. All SERS spectra collected with 1 s integration time, 785 nm laser excitation, and 1 mW power.

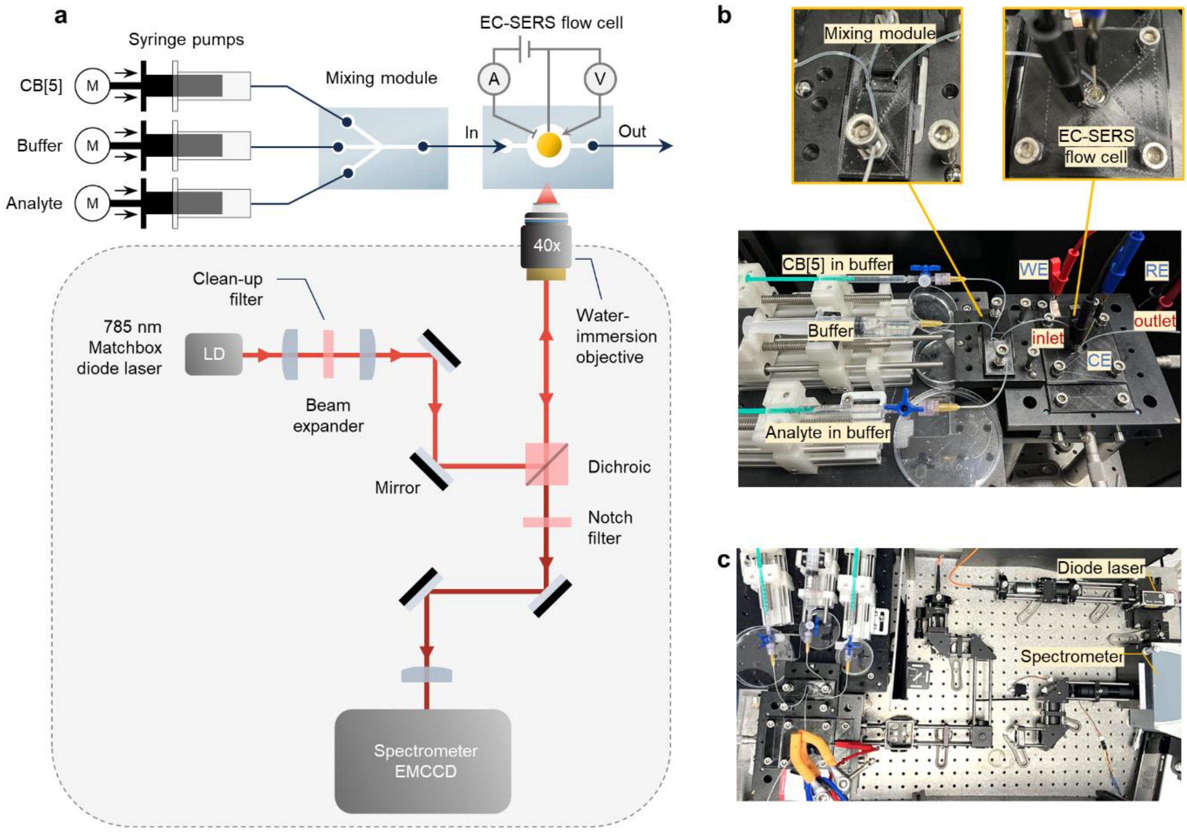

Supplementary Fig 4 | **Electrochemical SERS (EC-SERS) flow and optical set-up. a** Schematic diagram and photos of the **b** EC-SERS flow and **c** optical set-up.

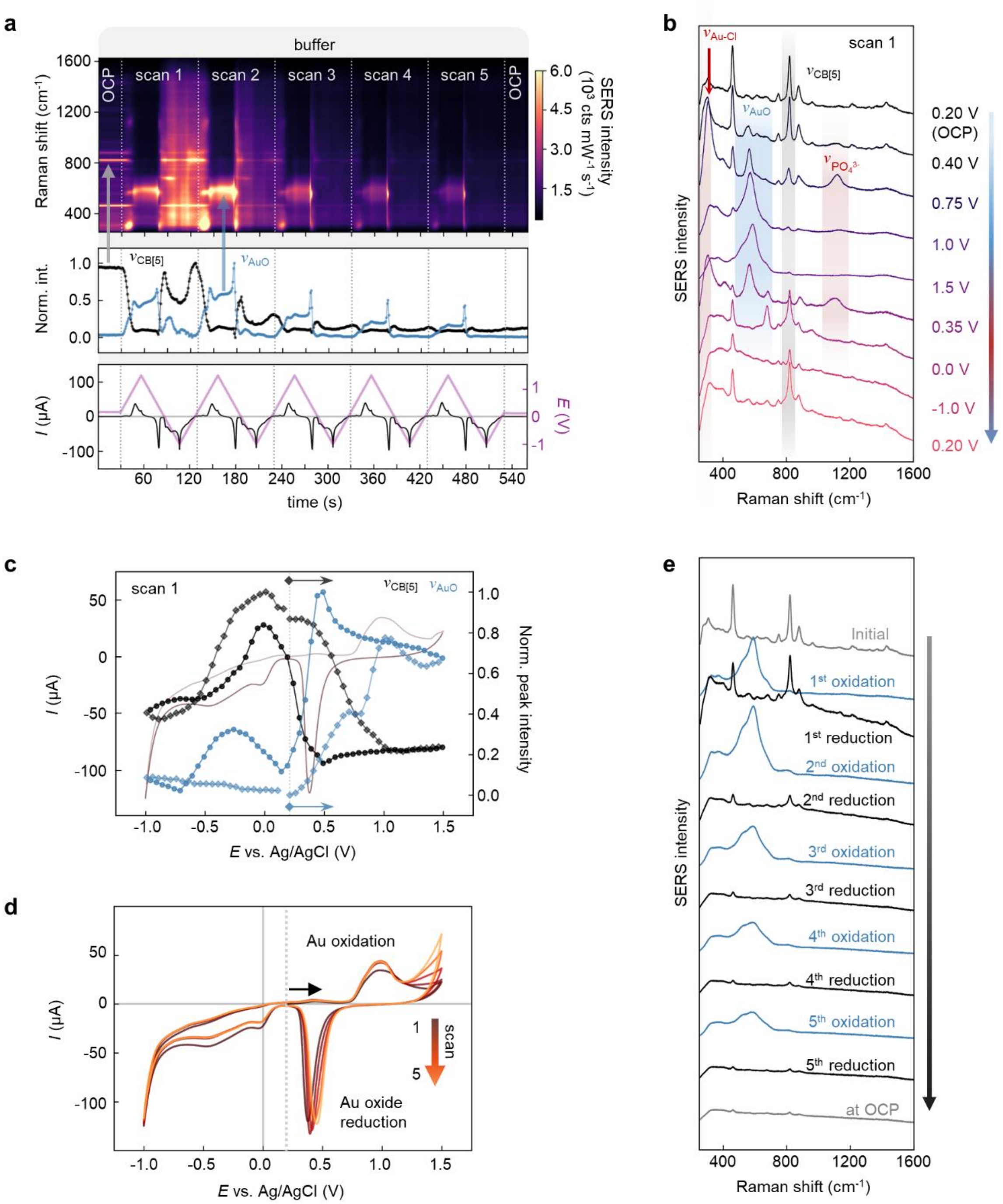

Supplementary Fig 5 | Cyclic voltammetry (CV) of MLagg-CB[5] in 50 mM potassium phosphate buffer. a Time-series SERS spectra (1 s integration time, 785 nm excitation laser with 1 mW power) from MLagg-CB[5] cycled between +1.5 V and -1.0 V in 50 mM potassium phosphate buffer (pH 7.0) at 50 mV s^{-1} for 5 scans. Intensities of CB[5] (830 cm^{-1}, black points) and Au-O peaks (broad peak centred at 590 cm^{-1}, blue points) are also plotted per SERS spectrum. The applied potential (purple solid line) and corresponding current response (black solid line) are plotted with time. **b** SERS spectra of the MLagg-CB[5] at various applied potentials during the first CV scan. **c** SERS spectro-voltammogram of the first CV scan, showing the evolution of CB[5] (black) and Au-O (blue) peak intensities as the potential is scanned, with diamond points indicating peak intensities as the potential is swept in the positive direction, whereas circles indicate peak intensities during potential sweeps in the negative direction. The dotted line indicates the applied starting potential. **d** Overlaid cyclic voltammograms from 5 scans. **e** SERS spectra of the MLagg-CB[5] with Au oxide (blue) and after the reduction of the oxide (black). Oxidised MLagg-CB[5] SERS spectra taken at +1.5 V, while reduced MLagg-CB[5] SERS spectra correspond to the spectra taken at +0.2 V after the competition of a full scan.

Supplementary Note 1 | Cyclic voltammetry of MLagg-CB[5]

CV in buffer

We first explore the changes in MLagg-CB[5] nanogaps under repeated anodic and cathodic polarisation using cyclic voltammetry (Supplementary Fig 5). SERS spectra are recorded every 1 s as the potential is swept at a scan rate of 50 mV s^{-1} in the positive direction from the open-circuit potential (OCP) of +0.2 V to +1.5 V, then in the negative direction to -1.0 V, and finally back to +0.2 V. Five scans are taken in 50 mM potassium phosphate buffer (pH 7.0). The peak intensities of features of interest are also tracked through SERS spectroscopy (Supplementary Fig 5a). These peaks include the characteristic CB[5] peak at 830 cm^{-1} ($\nu_{\mathrm{CB[5]}}$) which arises from the ring deformation vibration[2] and a group of peaks centred at 590 cm^{-1} (ν_{AuO}) originating from different types of Au-O bond stretching[3] in Au oxide and Au-OH.

At OCP, the MLagg SERS spectrum is dominated by the fingerprint of the CB[5] molecules scaffolding the nanogaps (Supplementary Fig 5b). As the potential is scanned in the positive direction, we first observe a current peak at ≈0.4 V, which can be attributed to the adsorption of anions[4]. The SERS spectra show increases in different peaks associated with anions: 1100 cm^{-1} for the electrolyte phosphate ions[5], 565 cm^{-1} for bound OH^-, and <300 cm^{-1} for bound residual chloride ions (likely from MLagg preparation). The rise in anion peaks is coupled with a decrease in CB[5] peak intensity, suggesting that at positive potentials (as the Au surface charge becomes increasingly more positive), the neutral molecular ligand, CB[5], is desorbed in favour of anions in the electrical double layer (EDL). It is also possible that at these positive potentials, CB[5] begins to oxidise.

At ≥ 0.75 V, we observe the start of an oxidative current as Au oxide formation begins. This process is coupled with the desorption of anions and the desorption and/or oxidation of CB[5]. By +1 V, both the oxidative current and ν_{AuO} intensity reach a maximum as all other species are desorbed and/or oxidised completely[4] (Supplementary Fig 5c). As the potential is further scanned to +1.5V, the ν_{AuO} intensity drops. This is consistent with previously reported SERS measurements during Au oxide formation: as thicker Au oxide layers are formed, a decrease in plasmonic coupling is observed as sub-surface nanogap Au atoms are oxidised to Au(III)[4]. From here, the potential is then swept in the cathodic direction. The ν_{AuO} intensity starts to rise again coupled with the start of a reductive current at +0.35 V. This sharp rise in ν_{AuO} peak intensity at the start of Au oxide reduction is consistent with the reduction of Au(III) back to Au atoms at the nanogap. However, as Au oxide reduction is complete, there is a sharp drop in ν_{AuO}, and a rise in the CB[5] peak and anion-related peaks. As the potential sweep is performed in static conditions, desorbed CB[5] molecules are likely sufficiently close to the MLagg surface to rebind once Au oxide is reduced. Further scans in the cathodic direction show the loss of remaining Au-O related peaks at 677 cm^{-1} and the desorption of anions. At -1 V, we observe a sharp reductive current, which corresponds to the hydrogen evolution reaction (HER). Sweeping the potential back to positive potentials leads to the re-adsorption of anions as the cycle is repeated.

Following the first potential scan, the SERS peak intensities of all features decrease by roughly half as the MLagg is oxidized and reduced during each scan (Supplementary Fig 5a,e). Changes in SERS intensity can either be associated with changes in the concentration of a species in the nanogap or changes in the SERS substrate enhancement factor. As the only CB[5] molecules in the system are those from the initial MLagg-CB[5] substrate, the decrease in CB[5] intensity can be attributed in part to its desorption and diffusion away from the nanogaps. However, the currents associated with Au oxide formation and reduction suggest that roughly similar amounts of Au are being oxidized and reduced in each scan (Supplementary Fig 5d). Thus, the gradual decrease in SERS intensity is likely due to the gradual degradation of the SERS hotspots.

CV in 1 mM CB[5] and buffer

As the MLAgg-CB[5] nanogaps degrade upon repeated Au oxidation and reduction, we investigate whether the hotspots can be stabilized in the presence of excess CB[5] in solution. The same potential scan is conducted on another MLagg-CB[5] substrate, but now with the addition of 1 mM CB[5]. The cyclic voltammograms and time-series SERS are shown in Supplementary Fig 6. While the CVs between the two MLagg-CB[5] samples are similar, with both samples exhibiting the characteristic Au oxidation, reduction, and HER currents (Supplementary Fig 5d and Supplementary Fig 6d), there are notable differences between the SERS observed with and without the presence of CB[5] in solution. After Au oxide is reduced back to Au, the CB[5] peak gradually returns close to its initial intensity, suggesting that CB[5] is rebinding to the Au surface to re-scaffold the nanogap hotspots after Au

oxide reduction. Thus, even after the first cycle, the gaps are stabilized so that reversible processes on the electrode such as Au oxidation/reduction are evident from the SERS spectra.

Despite the improved SERS stability, there is still a 10-20% decrease in SERS intensities after each oxidation and reduction cycle (Supplementary Fig 6a,c,e). We note that aside from the addition of CB[5] in the solution, there is also a concentration of chloride ions from the CB[5] reagent's acid of crystallisation. The presence of additional chloride ions in the electrolyte solution is evident from the SERS spectra: at OCP, a strong peak at <300 cm^{-1} (Supplementary Fig 6b) corresponds to specific adsorption of chloride ions to the Au surface[6]. The interaction of Cl^- with Au is very strong that even when its concentration is an order of magnitude lower than the phosphate buffer, its specific binding to Au is preferred over phosphate ions, as evidenced by the weaker phosphate ion peak intensities at 1100 cm^{-1} in Supplementary Fig 6b compared to those in Supplementary Fig 5b.

Another consequence of the presence of mM levels of Cl^- in solution is the formation of $[AuCl_4]^-$ surface complexes as Au is oxidized at anodic potentials. As the potential is scanned in the positive direction and Au oxide is formed in the presence of Cl^- ions in the electrolyte, a prominent new peak at 350 cm^{-1} appears and increases in intensity concurrently with the increase in ν_{AuO} (Supplementary Fig 6a,b,c). The 350 cm^{-1} peak originates from symmetric stretching of the Au-Cl bonds in $[AuCl_4]^-$. Since the $AuCl_4^-$ complex is water-soluble, this can lead to the excessive dissolution of Au atoms from the nanogap every time the MLagg is oxidised[7,8].

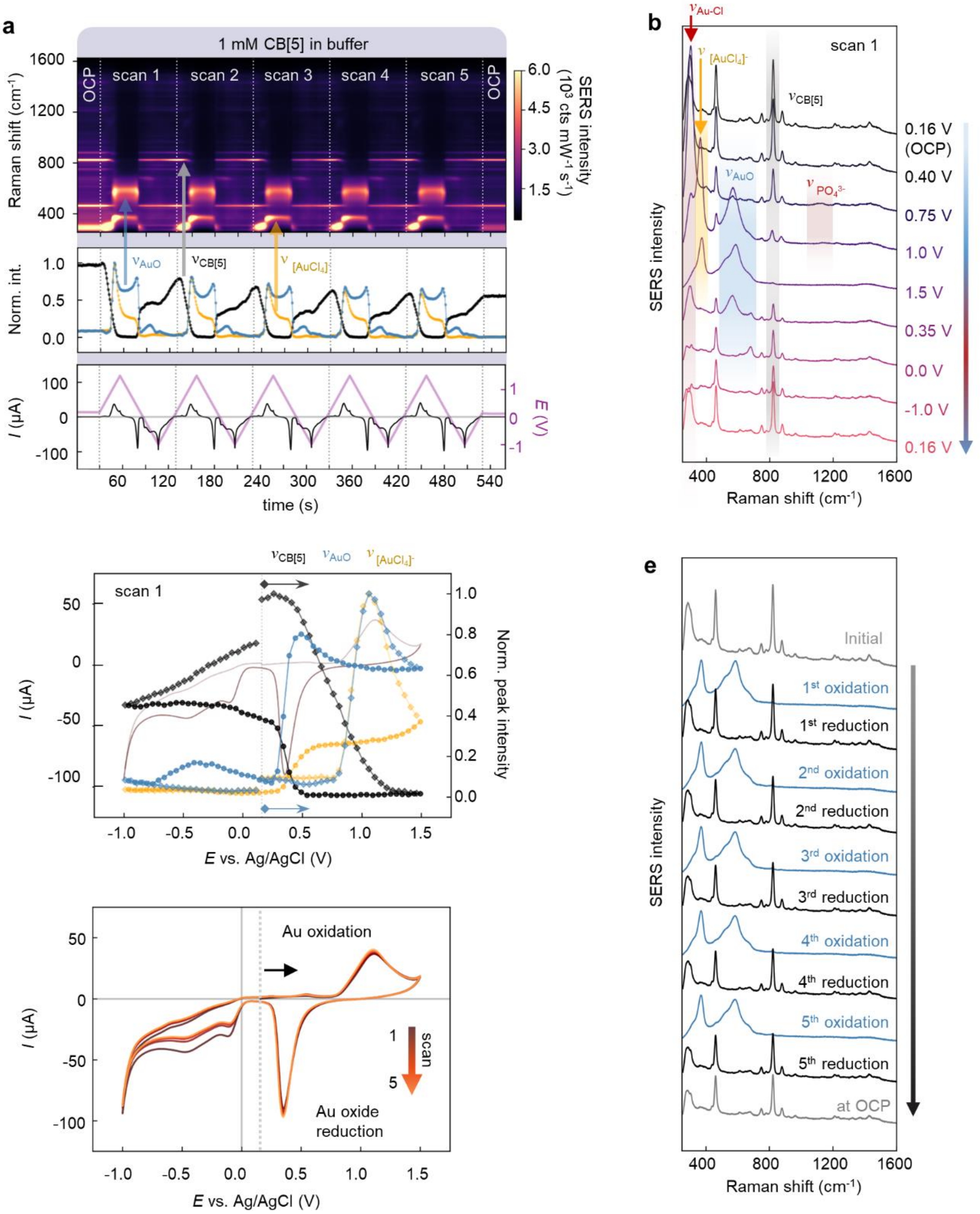

Supplementary Fig 6 | Cyclic voltammetry of MLagg-CB[5] in 1 mM CB[5] and buffer. a Time-series SERS spectra (1 s integration time, 785 nm excitation laser with 1 mW power) from MLagg-CB[5] cycled between +1.5 V and -1.0 V in 1 mM CB[5], 50 mM potassium phosphate buffer (pH 7.0) at 50 mV s^{-1} for 5 scans. Intensities of CB[5] (830 cm^{-1}, black points), Au-O peaks (broad peak centred at 590 cm^{-1}, blue points), and $[AuCl_4]^-$ (350 cm^{-1}, yellow points) are also plotted per SERS spectrum. The applied potential (purple solid line) and corresponding current response (black solid line) are plotted with time. **b** SERS spectra of the MLagg-CB[5] at various applied potentials during the first CV scan. **c** SERS spectro-voltammogram of the first CV scan, showing the evolution of CB[5] (black), Au-O (blue), and $[AuCl_4]^-$ (yellow) peak intensities as the potential is scanned, with diamond points indicating peak intensities as the potential is swept in the positive direction, whereas circles indicate peak intensities during potential sweeps in the negative direction. The dotted line indicates the applied starting potential. **d** Overlaid cyclic voltammograms from 5 scans. **e** SERS spectra of the MLagg-CB[5] with Au oxide present in the nanogaps (blue), and after the reduction of the oxide (black). Oxidised MLagg-CB[5] SERS spectra taken at +1.5 V, while reduced MLagg-CB[5] SERS spectra correspond to the spectra taken at +0.2 V after the competition of a full scan.

Supplementary Note 2 | Solution switching for oxidation and reduction of MLagg-CB[5]

To minimise the formation and possible dissolution of $[AuCl_4]^-$ from the nanogaps, the presence of Cl^- should be minimised during the oxidation step. We therefore investigate an alternative protocol in which the MLagg is oxidised in potassium phosphate buffer, while Au oxide reduction is conducted in the presence of CB[5]. Supplementary Fig 7a shows the time-series SERS spectra of a MLagg-CB[5] substrate undergoing an anodic linear scan from OCP (≈0.20 V) to +1.5 V in buffer, followed by a cathodic linear sweep from OCP to -1V and back to 0V in 1 mM CB[5] and buffer. Here, the 350 cm^{-1} peak corresponding to $[AuCl_4]^-$ is far less prominent than when oxidation is performed in the presence of excess CB[5] and Cl^- (Supplementary Fig 6). Reintroducing CB[5] during reduction allows the re-stabilisation of the nanogaps after Au oxide reduction.

We also explore another protocol using discrete potential steps (Supplementary Fig 8a). Here, the MLagg is oxidised with a potential step at +1.5 V in buffer, followed by a reducing potential step at -0.80 V in 1 mM CB[5] and buffer. With a potential step to +1.5 V, we observe a rapid drop in CB[5] peak with a rise in Au oxide peak within 1 s. Likewise, when switching the solution to 1 mM CB[5] and applying a potential step to -0.80 V, Au oxide is rapidly reduced with the re-adsorption of CB[5] at the nanogaps. The intensities of the SERS spectra in this case (Supplementary Fig 8b) are more consistent than when compared to the SERS spectra using slower potential sweeps (Supplementary Fig 7b). Since the oxidation and reduction steps are rapid, the possible dissolution of Au atoms is inhibited, resulting in reduced surface roughening[9].

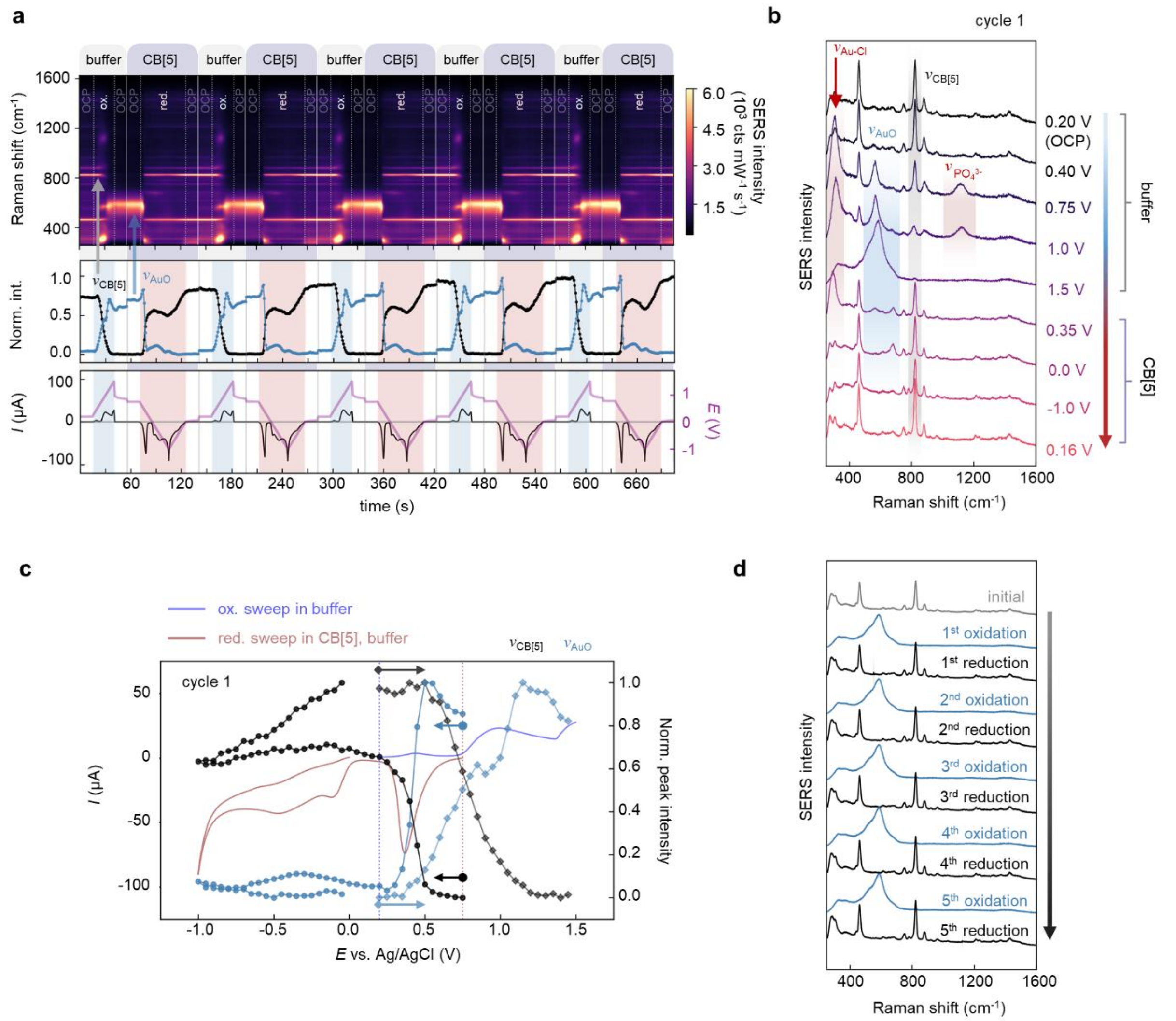

Supplementary Fig 7 | Linear sweep oxidation and reduction of MLagg-CB[5] in alternating solutions. a Time-series SERS spectra (1 s integration time, 785 nm excitation laser with 1 mW power) from MLagg-CB[5] undergoing an oxidising linear scan from OCP to +1.5 V in 50 mM potassium phosphate buffer (pH 7.0) at 50 mV s^{-1}, and a reducing linear scan from OCP to -1 V and back to 0 V in 1 mM CB[5] and buffer at 50 mV s^{-1} (total of 5 cycles). Intensities of CB[5] (830 cm^{-1}, black points) and Au-O peaks (broad peak centred at 590 cm^{-1}, blue points) are also plotted from each SERS spectrum. The applied or open-circuit potential (purple solid line) and corresponding current response (black solid line) are plotted with time. **b** SERS spectra of the MLagg-CB[5] at various applied potentials during the first cycle. **c** SERS spectro-voltammogram of the first oxidation/reduction cycle, showing the evolution of CB[5] (black) and Au-O (blue) peak intensities as the potential is scanned, with diamond points indicating peak intensities during the oxidising linear scan, whereas circles indicate peak intensities during the reducing linear scan. The blue and red dotted lines indicate the starting potentials of the oxidising and reducing scans, respectively. **d** SERS spectra of the MLagg-CB[5] with Au oxide (blue) and after the reduction of the oxide (black). Spectra are taken at OCP after each oxidising or reducing scan.

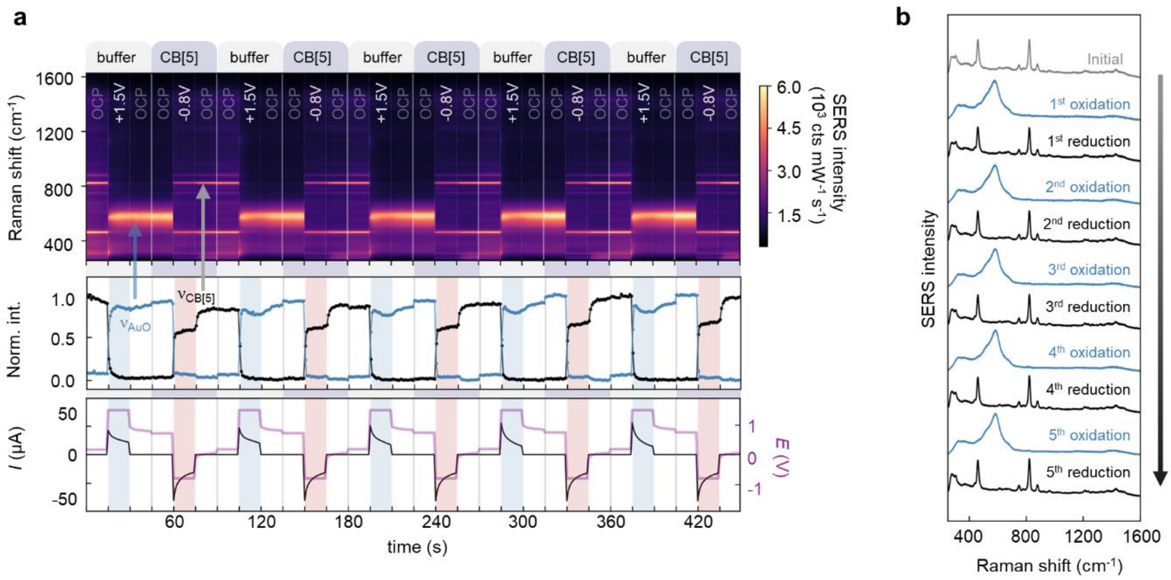

Supplementary Fig 8 | Potential step oxidation and reduction of MLagg-CB[5] in alternating solutions. **a** Time-series SERS spectra (1 s integration time, 785 nm excitation laser with 1 mW power) from MLagg-CB[5] undergoing an oxidising potential step to +1.5 V in 50 mM potassium phosphate buffer (pH 7.0), and a subsequent reducing potential step to -0.80 V in 1 mM CB[5] and buffer, for a total of 5 cycles. Intensities of CB[5] (830 cm^{-1}, black points) and Au-O peaks (broad peak centred at 590 cm^{-1}, blue points) are plotted from each SERS spectrum. The applied or open-circuit potential (purple solid line) and corresponding current response (black solid line) are plotted with time. **b** SERS spectra of MLagg-CB[5] with Au oxide (blue) and after the reduction of the oxide (black). Spectra are taken at OCP after each oxidising or reducing step.

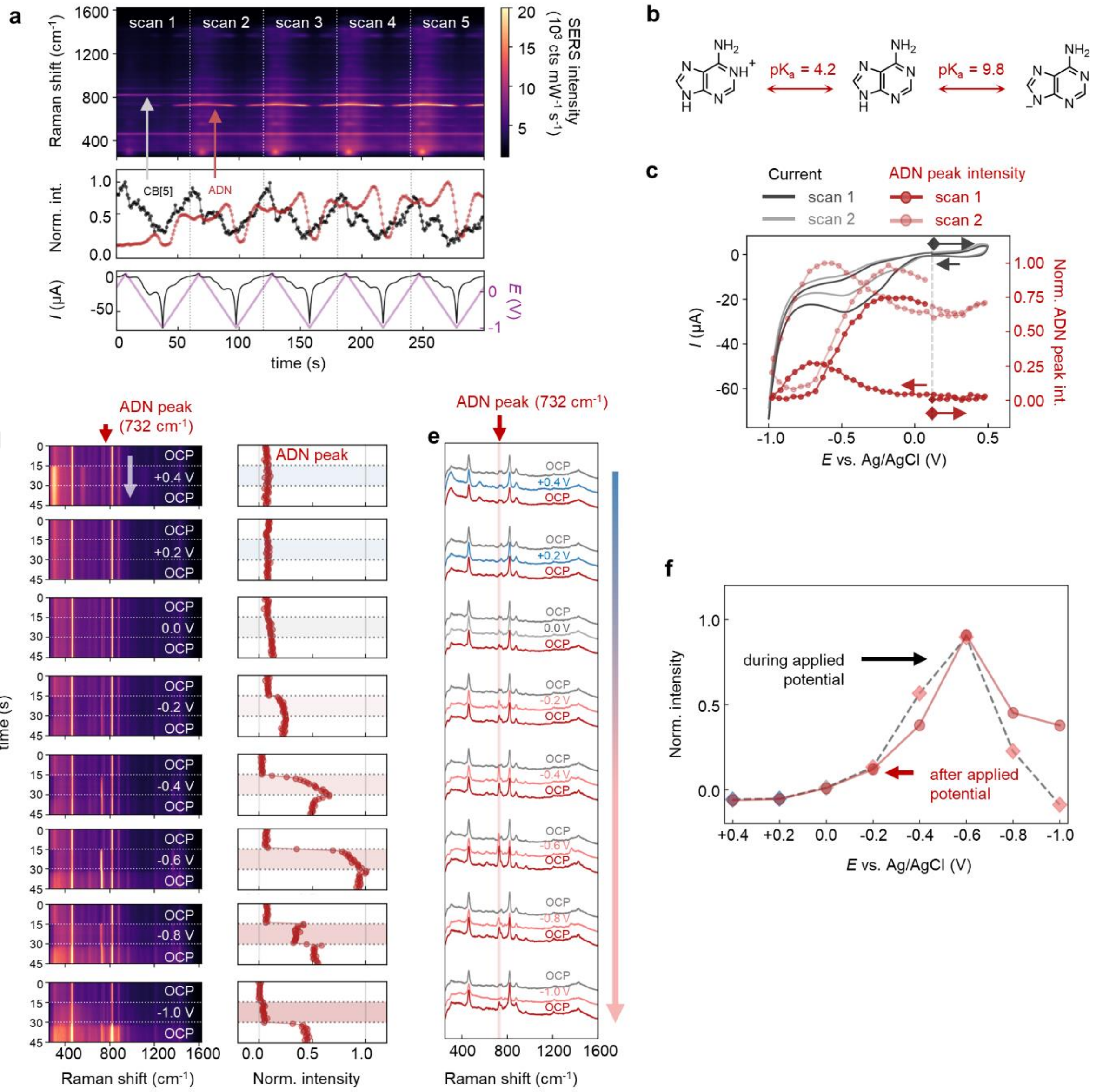

Supplementary Fig 9 | Potential-dependent binding of ADN on MLagg-CB[5]. a Time-series SERS spectra of the MLagg-CB[5] cycled between +0.5 V and -1 V in 10 μM adenine (ADN) and 50 mM potassium phosphate buffer (pH 7.0) at 50 mV s^{-1} for 5 scans. Peak intensities of CB[5] (830 cm^{-1}, black) and ADN (≈732 cm^{-1}, red) are also plotted per SERS spectrum. The applied potential (purple solid line) and corresponding current response (black solid line) are plotted with time. **b** Molecular structure of ADN and its acid and base forms. **c** SERS spectro-voltammogram of the CV scans 1-2, showing the evolution of ADN peak intensity (pink/red points) as the potential is scanned. Solid black line is the current response. The dotted line indicates the applied starting potential. **d** (*left*) Time-series SERS spectra of the MLagg-CB[5] incubated in 10 μM ADN in 50 mM potassium phosphate buffer (pH 7.0) at open-circuit potential (OCP), at various applied step potentials (vs Ag/AgCl), and after relaxation back to OCP. (*right*) ADN peak (ν_{ADN} ≈732 cm^{-1}) tracked from corresponding time-series SERS spectra. **e** SERS spectra before (*t* = 0 s from time-series SERS spectra), during (*t* = 30 s), and after different applied potentials (*t* = 45 s). **f** Normalised intensities of ADN peak during and after different applied potentials. All SERS spectra collected at 1 s integration time, 785 nm excitation laser with 1 mW power.

Supplementary Note 3 | Potential-dependent binding of ADN

We first investigate the effect of applied potentials on the SERS of a model analyte, adenine (ADN) using cyclic voltammetry. The MLagg-CB[5] is incubated in 10 µM ADN in 50 mM potassium phosphate buffer, and the potential is swept from the open-circuit potential (OCP ≈ 0.20 V) to +0.5 V at 50 mV s^{-1}. The scan direction is then reversed as the potential is swept in the negative direction to -1 V (Supplementary Fig 9a).

At pH 7.0, adenine is expected to be primarily neutral (Supplementary Fig 9b). At OCP, only a small ADN peak at 732 cm^{-1}, which corresponds to its totally symmetric ring breathing mode coupled with its in-plane NH_2 bend, is typically observed (Supplementary Fig 9a,c). Polarisation to positive potentials also results in only minor changes to the ADN peak, although at +0.4 V, we observe the appearance of new peaks at <300 cm^{-1} and 565 cm^{-1} corresponding to the binding of Cl^- and OH^-, respectively, at the nanogaps. At OCP and positive potentials, the electrical double layer (EDL) at the MLagg-CB[5] is dominated by adsorbed anions from the electrolyte, as the electrode surface is positively charged[10,11]. The layer of adsorbed anions and water molecules prevents more ADN from binding to the surface. However, as negative potentials are applied, anions start to desorb as the electrode passes its point of zero charge towards taking on a negative charge, where weakly bound, solvated cations dominate the inner layer of the EDL[10,11]. From here, ADN can more effectively compete for binding sites on the Au surface. Thus, the ADN peak increases after polarisation at negative potentials. In the first cycle, a maximum is reached at -0.60 to -0.70 V, followed by a decrease as the potential is further scanned to -1 V. However, scanning back to positive potentials shows an even greater increase in ADN peak intensity as the potential passes -0.60 V. Once the cycle repeats, polarisation to negative potentials results in further increases in ADN peak intensity (Supplementary Fig 9a,c), suggesting additional binding of ADN at the hotspots from the bulk solution as the potential is returned to a point that is optimal for ADN binding.

To investigate the possibility of rapidly enhancing the ADN signal by polarising the MLagg-CB[5] directly towards a potential that is optimal for ADN binding, we apply various step potentials starting from a clean MLagg-CB[5]. Here, a clean MLagg-CB[5] is first incubated in 10 µM ADN in buffer (pH 7.0) at open-circuit potential (OCP ≈0.2 V), followed by an application of a constant step potential for 15 s and relaxation back to OCP. SERS spectra are recorded every 1 s before, during, and after the applied potential (Supplementary Fig 9d-e). Consistent with the CV-SERS measurements, potential steps to positive potentials show only minor changes to the ADN peak (Supplementary Fig 9d-e), while negative potentials show rapid increases in ADN signal. The intensity of this peak reaches a maximum at by holding the potential at -0.60 V and gradually decreases at more negative potentials (Supplementary Fig 9d).

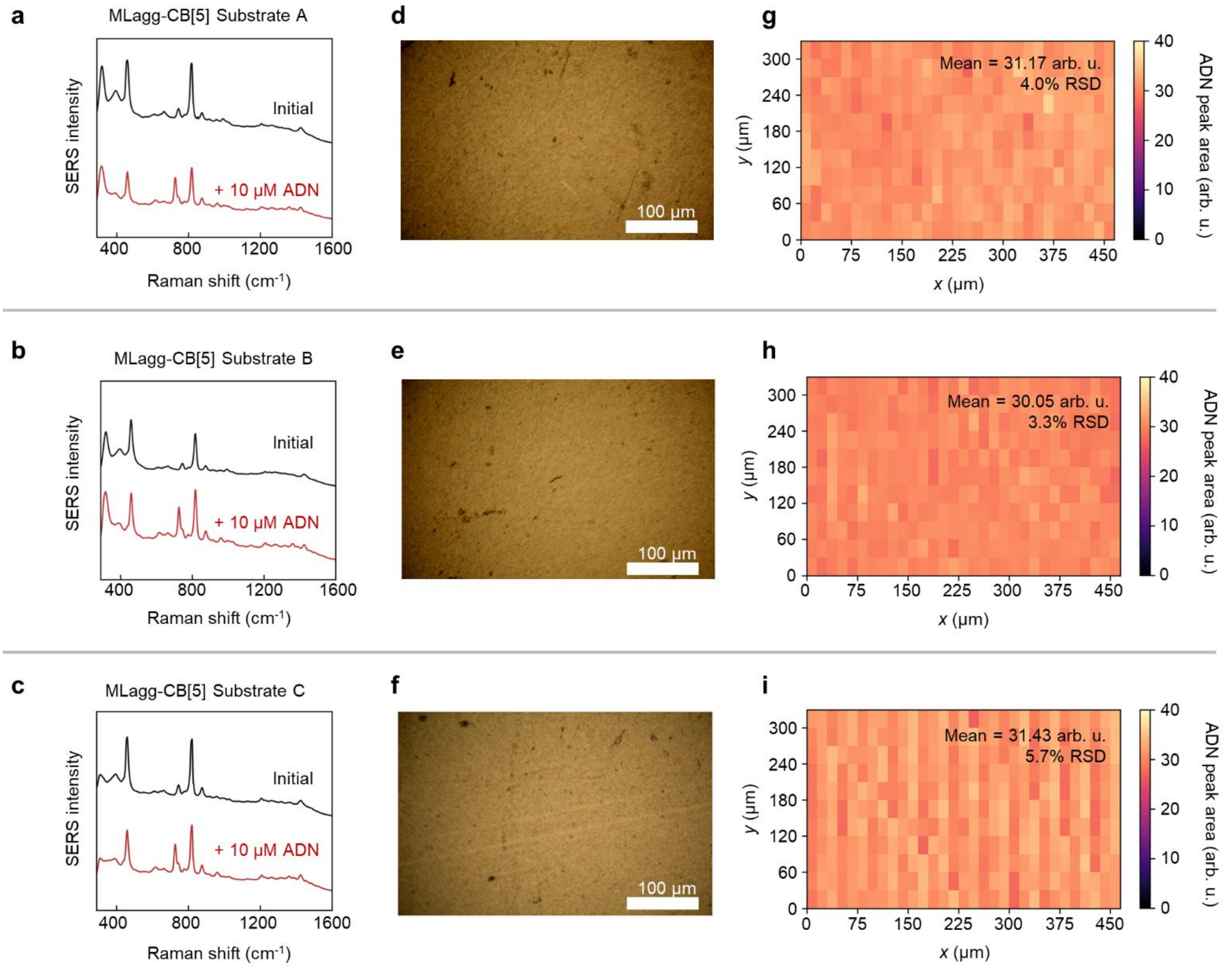

Supplementary Fig 10 | Regional uniformity of ADN signal over different MLagg-CB[5] substrates. For three different MLagg-CB[5] substrates: **a, b, c** SERS spectra of MLagg-CB[5] before (black) and after (red) 10 µM ADN binding with -0.60 V enhancement potential in 50 mM potassium phosphate buffer (pH 7.0). Spectra were taken *in-situ* with 1 s integration time, 785 nm laser with 1 mW power using a 40x objective. **d, e, f** Optical microscope image (465 x 330 µm region) of rinsed and dried MLagg-CB[5] surface after 10 µM ADN binding, and the **g, h, i** corresponding heatmap of SERS ADN peak area (ν_{ADN} = 732 cm^{-1}) over the MLagg-CB[5] surface area taken over a 31 x 11 grid with 15 x 30 µm spacings. SERS map captured using 1 s integration time, 785 nm excitation laser, 2.14 mW power with a 20x objective.

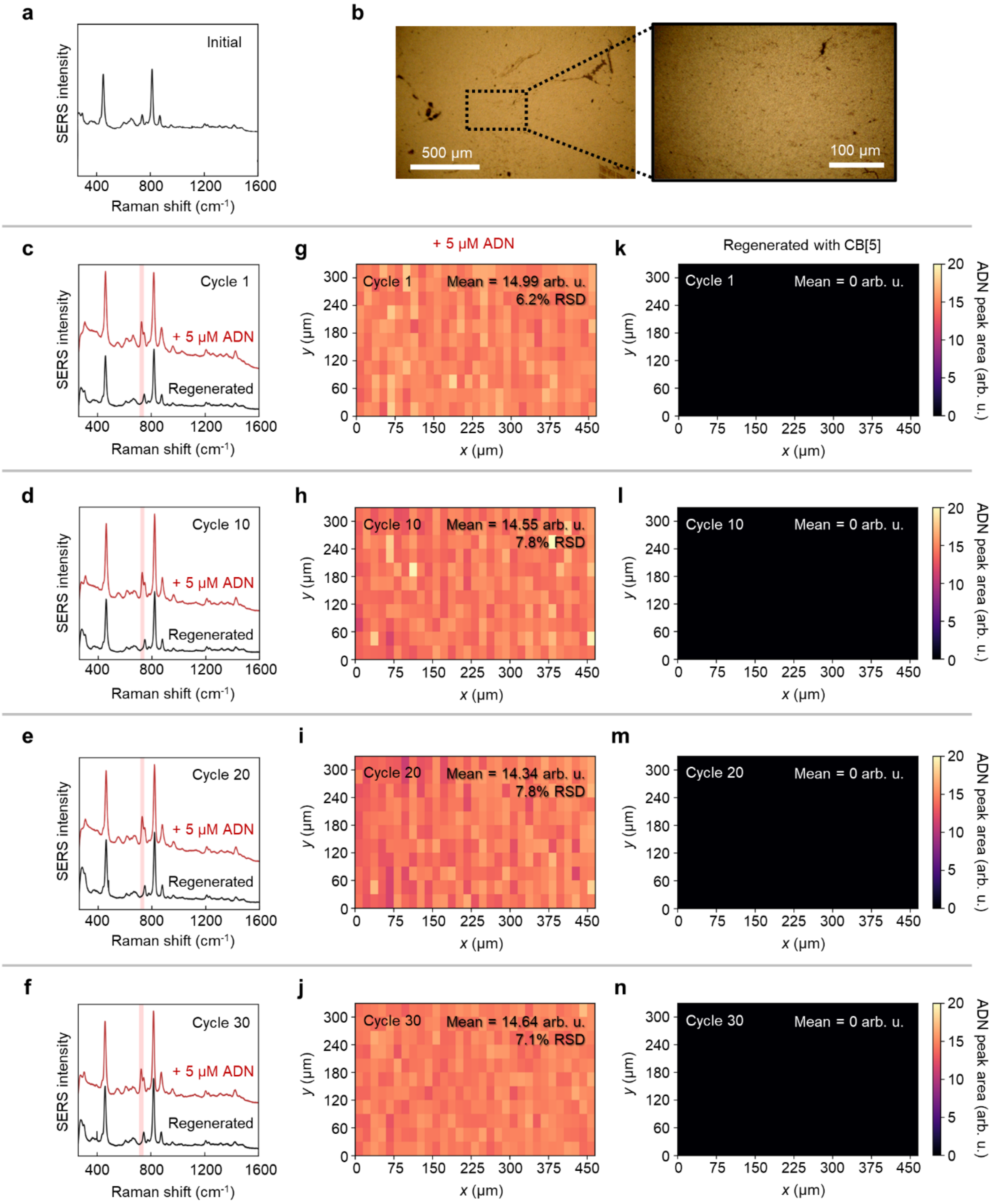

Supplementary Fig 11 | Regional uniformity of ADN signal on MLagg-CB[5] over multiple cycles of analyte detection and cleaning/regeneration with CB[5]. a SERS spectrum of MLagg-CB[5] before analyte cycling tests. **b** Optical microscope image of 465x330 µm region-of-interest used for SERS mapping. For cycles 1, 10, 20 and 30: **c, d, e, f** SERS spectra of MLagg-CB[5] after 5 µM ADN binding with -0.60 V enhancement potential in 50 mM potassium phosphate buffer (pH 7.0) (red), and after cleaning and regeneration with CB[5] (black). Spectra were taken *in-situ* with 1 s integration time, 785 nm laser with 1 mW power using a 40x objective. Heatmaps of SERS ADN peak area (ν_{ADN} = 732 cm^{-1}) over the dried MLagg-CB[5] surface area **g, h, i, j** after ADN binding and **k, l, m, n** after ReSERS with CB[5]. Heatmaps were taken over the region shown in **b** on a 31x11 grid with 15x30 µm spacings. SERS map captured using 1 s integration time, 785 nm excitation laser, 2.14 mW power with a 20x objective.

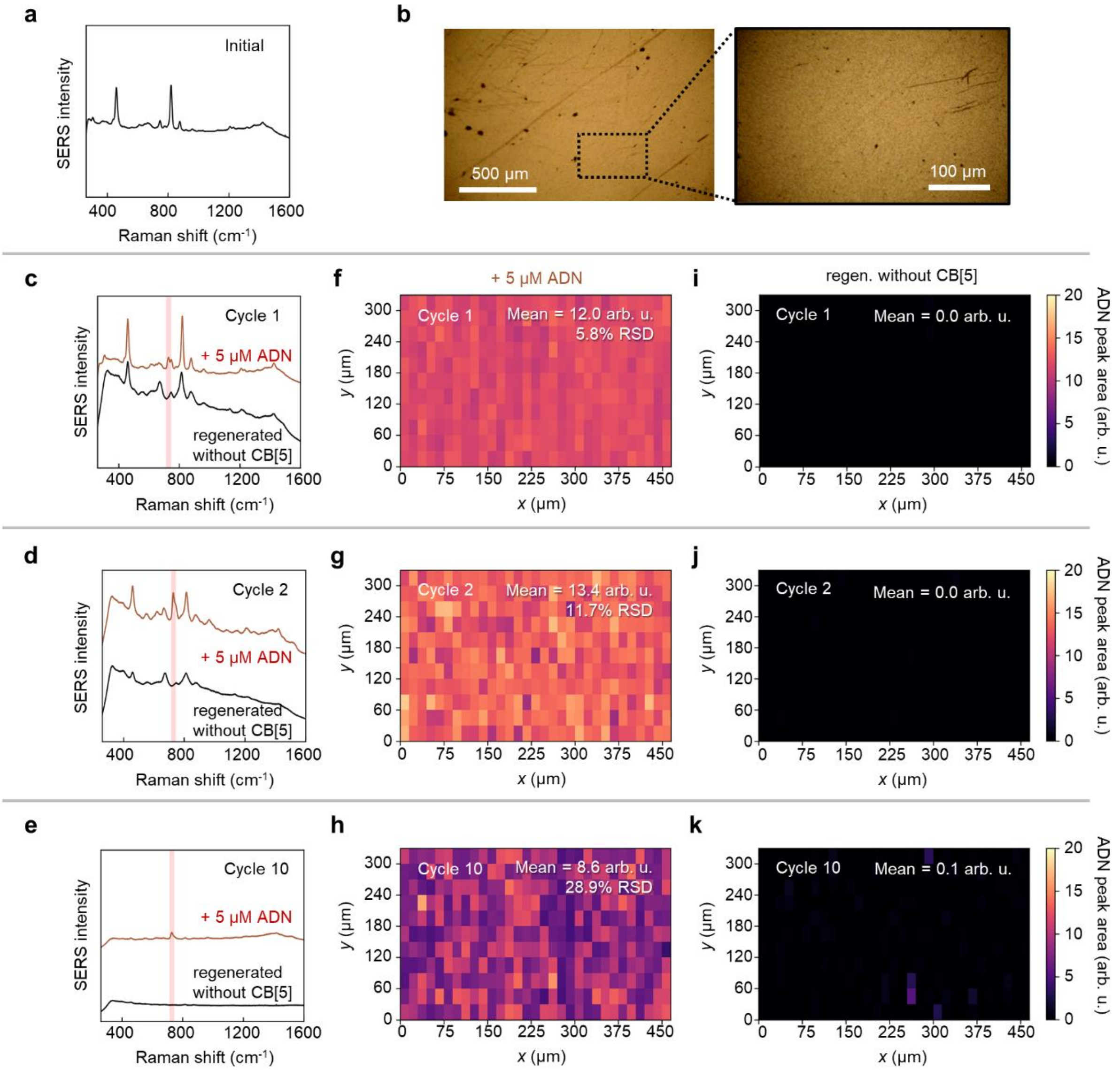

Supplementary Fig 12 | Regional uniformity of ADN signal on MLagg-CB[5] over multiple cycles of analyte detection and cleaning/regeneration without CB[5]. a SERS spectrum of MLagg-CB[5] before analyte cycling tests. **b** Optical microscope image of 465x330 µm region-of-interest used for SERS mapping. For cycles 1, 2 and cycle 10: **c, d, e** SERS spectra of MLagg-CB[5] after 5 µM ADN binding with -0.60 V enhancement potential in 50 mM potassium phosphate buffer (pH 7.0) (red), and after cleaning and regeneration without CB[5] (black). Spectra were taken *in-situ* with 1 s integration time, 785 nm laser with 1 mW power using a 40x objective. Heatmaps of SERS ADN peak area (ν_{ADN} = 732 cm^{-1}) over the dried MLagg-CB[5] surface area **f, g, h** after ADN binding and **i, j, k** after ReSERS without CB[5]. Heatmaps were taken over the 465x330 µm region shown in (b, right) on a 31x11 grid with 15x30 µm spacings. SERS map recorded using 1 s integration time, 785 nm excitation laser, 2.14 mW power with a 20x objective.

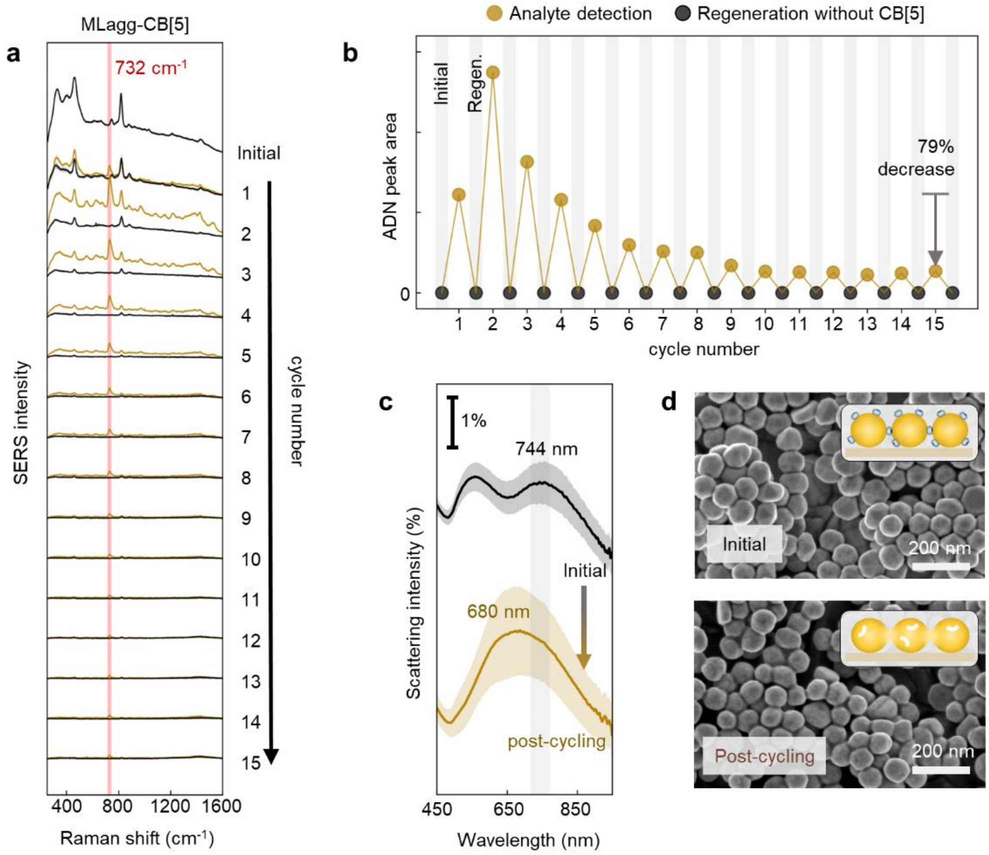

Supplementary Fig 13 | Cycles of 10 µM ADN detection, cleaning and regeneration with buffer and 1 mM KCl on MLagg-CB[5]. **a** SERS spectra from 15 cycles of 10 µM ADN detection (yellow) and cleaning/regeneration in 1 mM KCl, 50 mM potassium phosphate buffer, pH 7.0 (black). Spectra are offset for clarity. **b** ADN peak areas after analyte detection and cleaning/regeneration. **c** Dark field scattering spectra and **d** scanning electron micrographs of the MLagg-CB[5] before and after 15 cycles and analyte detection and cleaning/regeneration in 1 mM KCl and buffer. For the DF spectra in **c**, solid lines and shaded area represent mean and ±1 s.d. of *n*=150 spectra obtained across the area of a MLagg-CB[5]. Grey line highlights initial chain mode peak wavelength. Spectra are offset for clarity.

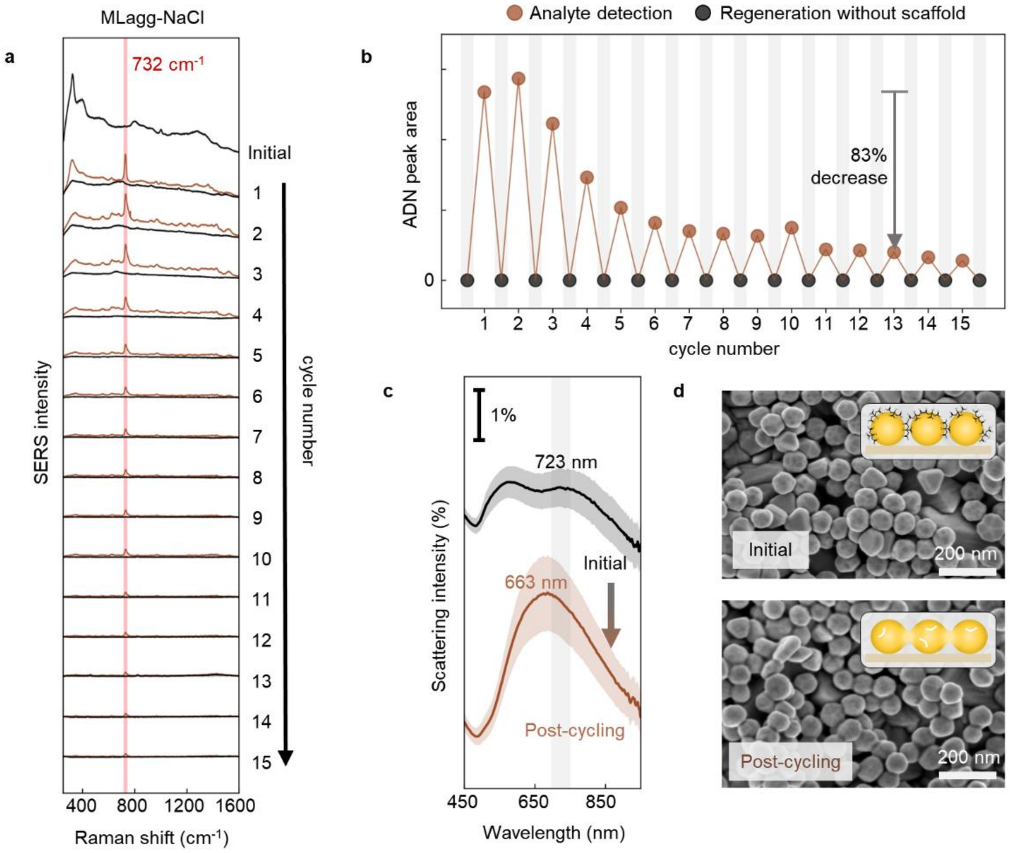

Supplementary Fig 14 | Cycles of 10 µM ADN detection, cleaning, and regeneration with buffer on MLagg-NaCl. a SERS spectra from 15 cycles of 10 µM ADN detection (brown) and cleaning/regeneration in 50 mM potassium phosphate buffer, pH 7.0 (black). Spectra are offset for clarity. **b** ADN peak areas after analyte detection and cleaning/regeneration. **c** Dark field scattering spectra and **d** scanning electron micrographs of the MLagg-NaCl before and after 15 cycles and analyte detection and cleaning/regeneration in buffer. For the DF spectra in **c**, solid lines and shaded area represent mean and ±1 s.d. of *n*=150 spectra obtained across the area of a MLagg-CB[5]. Grey line highlights initial chain mode peak wavelength. Spectra are offset for clarity.

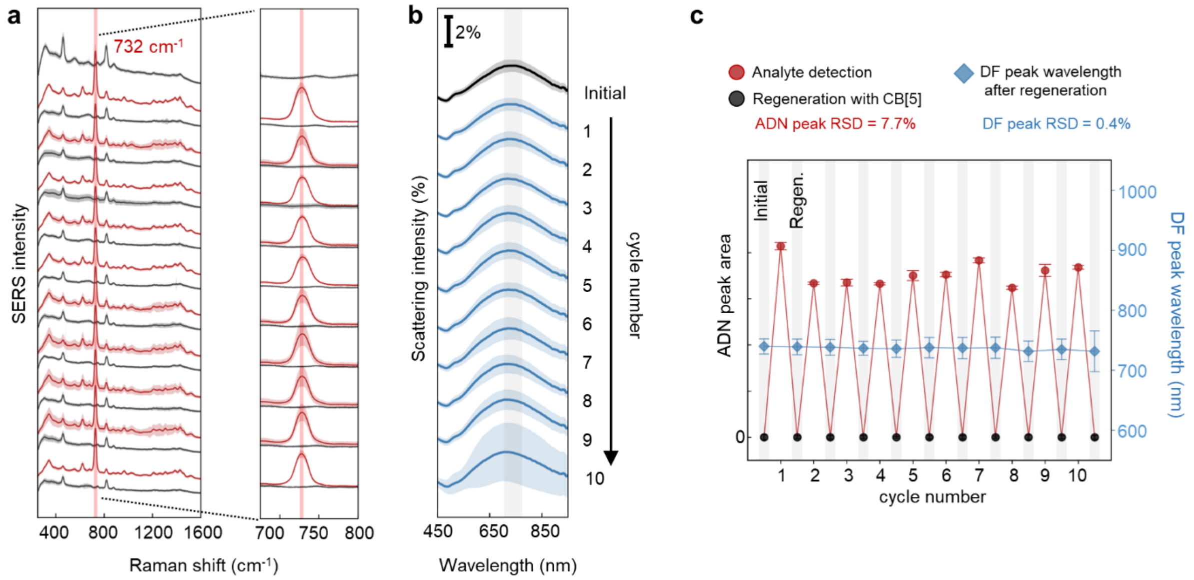

Supplementary Fig 15 | Cycles of 100 µM ADN detection, cleaning, and regeneration with CB[5] on MLagg-CB[5] with DF measurements at each regeneration step. a SERS spectra from 10 cycles of 100 µM and detection (red) and cleaning/regeneration with CB[5] (grey). Solid line and shaded region represent the mean and ±1 s.d. of spectra each captured over *n*=10 different points across the MLagg-CB[5] surface area. **b** DF scattering spectra of the MLagg-CB[5] before (black) and after (blue) cleaning/regeneration with CB[5] over 10 cycles. Solid lines and shaded area represent mean and ±1 s.d. of spectra each obtained across *n*=150 points the area of the MLagg-CB[5]. **c** ADN peak areas (determined from spectra obtained from *n*=10 points across the MLagg-CB[5] surface area; error bars represent ±1 s.d.) after analyte detection (red) and cleaning/regeneration (black). Mean DF peak wavelengths of each regenerated MLagg-CB[5]. Error bars give ±1 s.d. from *n*=150 DF scattering spectra.

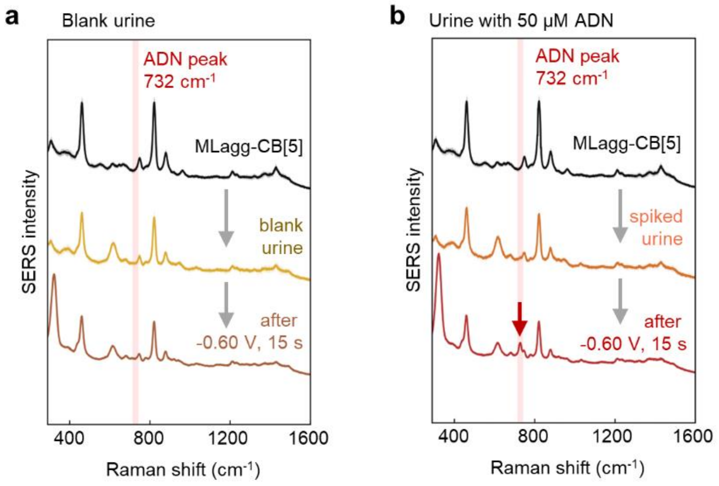

Supplementary Fig 16 | SERS spectra from blank and spiked urine test samples. a SERS spectra from the MLagg-CB[5] in buffer (black), blank urine matrix (no ADN added, yellow), and after the application of -0.60 V enhancement potential (brown). **b** SERS spectra from the MLagg-CB[5] in buffer (black), spiked urine matrix (with 50 µM ADN added, orange), and after the application of -0.60 V enhancement potential (red). Red arrow points to characteristic ADN peak at ν_{ADN}=732 cm^{-1}

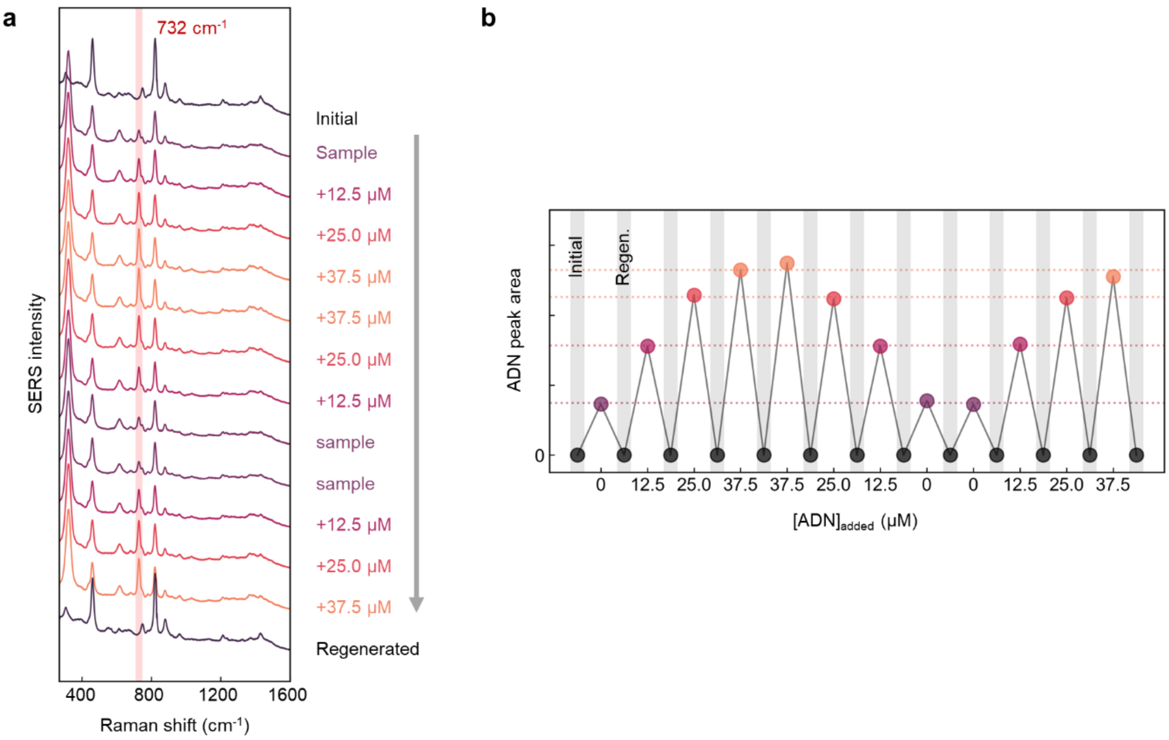

Supplementary Fig 17 | Standard additional analysis of ADN in urine. a SERS spectra from the sequential analysis of ADN standards. **b** ADN peak areas from the SERS spectra of sequentially measured ADN standards and the corresponding regenerated MLagg-CB[5] (black). Dotted horizontal lines represent the average peak areas of each ADN standard.

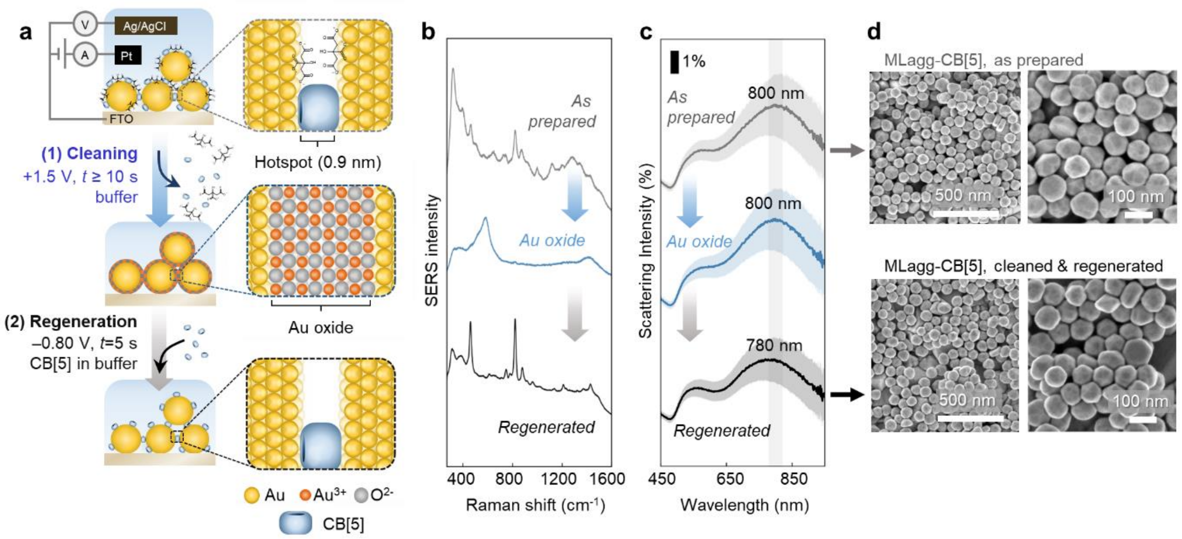

Supplementary Fig 18 | Initial EC cleaning and regeneration of MLagg-CB[5]. a Schematic of the initial cleaning and regeneration of freshly prepared MLagg-CB[5] using *in situ* electrooxidation and reduction. **b** SERS spectra and **c** DF scattering spectra from the MLagg-CB[5] as prepared (grey), after electrooxidation (blue), and after CB[5]-regeneration (black). For the DF spectra in **c**, solid lines and shaded area represent mean and ±1 s.d. of n=150 spectra obtained across the area of a MLagg-CB[5]. Grey line highlights initial chain mode peak wavelength. Spectra are offset for clarity. **d** Scanning electron micrographs of the MLagg-CB[5] (*top*) as prepared and (*bottom*) after initial cleaning and regeneration.

Supplementary Table 1 | Studies reporting different strategies for SERS substrate recycling or cleaning. (Asterisk indicates that % RSD estimated from reported data)

Type	Ref	SERS Substrate	Sample Analysis				Recycling Method					All steps *in situ*?	Remarks
			Protocol	*Analytes*	*LOD*	*%RSD*	*Protocol*	*Cleaning Time*	*# cycles*	*Repeatability between cycles*	*Removed analytes*		
Works reporting chemical etching and metal redeposition for SERS substrate renewal													
Chemical etching and template-based renewal	12	3D-nanostar-dimer-in-ring structure (3D-NSDiRs), where AuAg layer is deposited on an underlying Si substrate	Immersion in analyte solution for up to 24 hrs, followed by rinsing and drying	p-amino-thiophenol (pATP), rhodamine 6G (R6G), and adenine	≈ pM	n.r.	Removal of analyte and metal coating by chemical etching (KI/I_2) and plasma cleaning, followed by thermal evaporative redeposition of Ag and Au	n.r. (not reported)	5	< 10% RSD	10 µM pATP	no	Requires clean room facilities and handling of caustic reagents for chemical etching
Chemical etching and template-based renewal	13	AuNPs that can slide and aggregate on recyclable alumina/silicon nanohoodoos [10^7-10^8 EF]	Incubation in analyte solution, followed by drying of solvent, which facilitates aggregation of AuNPs	trans-1,2-bis (4-pyridyl) ethylene (BPE) in EtOH	≈pM	6% CV (substrate uniformity)	aggregated AuNPs are etched from the nanohoodoos via $KI/I_2/H_2O$ bath, followed by re-deposition of fresh Au via e-beam evaporation	n.r.	8	≈ 3% RSD*	1 nM trans-1,2-bis (4-pyridyl) ethylene (BPE)	no	Requires clean room facilities and handling of caustic reagents for chemical etching
Chemical etching and template-based renewal	14	Klarite	Analyte solution drop-cast onto substrate and allowed by air dry	Benzoic acid in EtOH	≈µM	10-15% (substrate uniformity)	Removal of analyte and Au surface by chemical etching (aqua regia) and piranha solution. Au redeposited with vacuum evaporator	n.r.	4	14-31% RSD	Benzoic acid	no	Requires handling of caustic reagents for chemical etching
Works reporting analyte degradation via thermal, plasma, or photochemical treatment													
Thermal decomposition	15	Au/Ag bi-layer film with protective alumina coating [5.5 x 10^4 EF]	Aqueous analyte solution is drop-cast onto substrate and evaporated at 100°C	n.r.	n.r.	19% (substrate uniformity)	thermal treatment for 5 min at 400°C to decompose analyte, followed by cooling to room temperature before next measurement	5 min	5	15% decrease in signal from cycle 1 to 5	0.1 mM R6G	no	Can remove most organic compounds, as most have decomposition points below 400°C. Inorganic contaminants may remain
Thermal decomposition	16	Sputtered AuNPs on boron nitride nanosheets	Incubation in analyte solution for 1 hr, followed by rinsing and drying	n.r.	n.r.	n.r.	thermal treatment for 5 min at 400°C to decompose analyte	5 min	5	≈14% RSD*	1 µM R6G	no	
Thermal decomposition	17	graphene-covered Au or Ag triangular nanoarrays (TNAs)	Incubation in analyte solution followed by drying	R6G, methylene blue, amoxicillin	≈0.1 µM R6G (dye)	n.r.	Thermal treatment for 30 min at 300°C under protective atmosphere of Ar	30 min	16	n.r. analyte signal halved after first cycle; and continuously decreases	10 µM R6G, methylene blue	no	After >15 cycles, graphene coating thermally damaged
Plasma cleaning	18	Au-coated tungsten nanofuzz array (TNFA)	Incubation in sample for 1 minute, followed by drying in air	Rhodamine B (RhB), brilliant blue (X-BR) and reactive brilliant red (X-3B)	≈ppb RhB (dye)	n.r.	atmospheric pressure plasma treatment for 5 min (He with gas flow rate of 1 SLM and at an alternating voltage of 20 kV)	5 min	5	≈40% RSD*	10 ppb RhB	no	
Plasma cleaning (initial cleaning)	19	Ag nanorod arrays prepared from oblique-angle vapor deposition	Incubation in solution for 12 hrs, followed by rinsing and drying	n/a	n.r.	n.r.	Ar plasma cleaning (100 W, 1-2 SCFH Ar flow)	4 min	n.r.	n.r. SERS signal halves after first cleaning	1,2-bis(4-pyri-dyl)ethylene (BPE) 1-propanethiol in EtOH	no	Longer Ar plasma cleaning times resulted in greater morphological changes, and decrease in SERS activity by factor of 10
Plasma cleaning	20	Au-based multistack vertically oriented nanogap hotspots in nanolaminated plasmonic crystals (8 nm nanogaps) [10^7-10^8 EF]	Incubation in analyte solution for 24 hrs followed by ethanol rinsing and drying	benzenethiol in ethanol	n.r.	28.12%	O_2 plasma cleaning (50W, 50 sccm O_2 gas flow)	40 s	4	Uniformity of substrate degrades from 38% to 69% to 62%	benzenethiol in ethanol	no	

Plasma cleaning	21	Au/Pd-coated chestnut-like copper oxide film [10^4-10^5 EF]	Analyte solution drop-cast onto substrate and allowed by air dry	R6G in EtOH	n.r.	n.r.	O_2/Ar mixture plasma cleaning (100W, 160 sccm Ar, 40 sccm O_2 gas flows)	50 s	5	≈11% RSD*	R6G	no	
Photochemical degradation	22	AuNP-decorated silicon nanowire array (Au-SiNWA)	Incubation in analyte solution for 5 hrs	rhodamine B (RhB)	10^{-11} M RhB (dye)	n.r.	Substrate irradiated for 180 minutes under UV (UV light photocatalytic degradation), followed by rinsing and drying at 60°C under vacuum	180 min	4	n.r.	1 µM RhB	no	
Photochemical degradation	23	Fe_3O_4@SiO_2@TiO_2@Ag (Multi-functional microspheres - magnetic and photocatalytic and SERS active)	dispersed microspheres incubated with analyte, collected magnetically and deposited on solid support for SERS analysis dry.	R6G	10 ppb R6G (dye)	5%	Redispersion of microspheres in water, followed by 100 min exposure under UV irradiation and DI water rinsing	100 min	7	n.r.	R6G	no	
Photochemical degradation	24	Colloidal PbS-Au hybrid [5.1 x 10^8 AEF]	sample and colloidal SERS substrates mixed and incubated for 2 hrs in dark. Suspension then drop-cast on solid support and dried at 70°C for 2 hours.	RhB	10^{-12} M RhB (dye)	n.r.	Visible light irradiation (300 W Xe lamp)	90 min	10	Decrease in SERS signal by 17% from cycle 1 to 10	RhB	no	
Photochemical degradation	25	CuO/Ag composite film	substrate soaked in analyte solution for 20 min to 20 hrs, followed by rinsing with DI water or EtOH, and drying in air	R6G, crystal violet, thiram	10^{-8} M thiram	8.9% (uniformity)	Substrate immersed in DI water and irradiated with visible light irradiation (300 W Xe lamp)	100 min	3	n.r.	1 µM R6G	no	
Photochemical degradation	26	TiO_2/Ag long-range ordered crystals	Incubation in analyte solution for 1 hr in the dark	RhB	10^{-7} M RhB (dye)	n.r.	Exposure to LED light (370 nm), followed by DI water rinsing and drying with N_2 gas	120 min	3	≈ 5% RSD*	1 mM RhB	no	
Photochemical degradation	27	Au semishells on TiO_2 spheres	Incubation in analyte solution for 30 min, rinsed with DI water, and dried with N_2	R6G, brilliant cresyl blue (BCB)	10^{-9} M R6G (dye)	12% (uniformity)	Immersed in DI water and irradiated with 4W UV lamp (254 nm), followed by rinsing with DI and drying with N_2	1.5-2 hrs	5	n.r.	1 µM R6G, 0.1 µM BCB	no	
Photochemical degradation	28	4N-in-1 hybrid substrate (Ag–TiO_2) [50x EF]	Solution drop-cast onto SERS substrate	R6G	≈10^{-14} M R6G (dye)	n.r.	Exposure of substrate to high intensity (10 mM-cm^{-2}) UV light	3-25 min	10	≈ 5% RSD*	10^{-15} – 10^{-5} M R6G; Toluene, allura red, erythrosine, indigo tine, Brown HT	possible	Required illumination time dependent on analyte concentration; quoted number of cycles is 18, but data demonstrates 10 detection/cleaning cycles
Works that report the use of special polymer coatings to modulate analyte binding/removal													
Thermally-responsive polymer coating	29	Au@pNIPAM (thermally responsive poly-(N-isopropylacrylamide))	Analyte incubated for 2 hrs with Au@pNIPAM in swollen state (at 4°C), to retain analyte in polymer. Polymer collapses upon heating to 60°C for 2 hrs, brings analyte closer to Au.	n.r.	n.r.	n.r.	Au@pNIPAM is cooled back to 4°C for 2 hrs to swollen state so that analyte can be released from Au surface.	At least 2 hrs	n.r.	n.r.	1-napthol, 1-naphtalenethiol (1NAT), Nile Blue A	possible	
Thermally-responsive polymer coating	30	NP-on-mirror (AuNP on Au/SiO_2-coated Si optical interference substrate) with thiolated pNIPAM polymer coating serving as molecular trap	Immersion in analyte solution with heating to 50°C. System cooled to 4°C and washed 5x to remove excess analyte.	R6G	10^{-9} M R6G (dye)	15.7% CV uniformity; 5.6% CV substrate reproducibility	Sample is exposed to hot oxalic acid solution (50°C) for 10 minutes and washed with hot oxalic acid solution 5x. Sample is then taken out from solution and dried immediately	At least 10 min	5	≈ 5% RSD*	100 µM R6G	no	
Thermally-responsive polymer coating	31	poly(N-isopropyl acrylamide) (PNIPAm)-grafted gold grating	Incubation in analyte solution at room temperature (24°C) for 15-30 min, followed by heating for 5	crystal violet (CV), disperse red 1(DR1),	≈10^{-14} M CV (dye)	3% CV (uniformity), 4.5%	Analyte removed by immersing substrate in DI for 15 min at room temperature	15 min	3	5% RSD	10^{-14} M CV, DR1, ME	no	

			min, washing 3 times with hot water, and drying under air	and metanil yellow (ME)		substate reproducibility						
Thermally-labile polymer coating	32	AuNP superlattice spin-coated with thermally labile poly(lactic-co-glycolic acid) (PGLA) layer	Windows exposed on the protective PGLA layer by locally irradiating a spot with 0.064 mW μm^{-2} for 1 s, allowing analyte from solution to bind to exposed SERS substrate. Measurement taken after 5 s.	n.r.	n.r.	n.r.	For new sample analysis, a new spot on the substrate is exposed by selectively degrading the protective PGLA layer	At least 1s for new window	11	≈ 26% RSD* for TBZ (4 cycles)<br>≈ 20% RSD* for MBA (3 cycles)	4-mercaptobenzoic acid (4-MBA), nicotinamide (NAm), methylene blue, thiabendazole, CV, adenosine	yes	10,000 consecutive measurements per substrate is theoretically possible; Reproducibility of analyte signals per cycle dependent on underlying SERS substrate
Works that report removal of adsorbates via solution/organic solvent rinsing												
Immersion	33	Flower-like Ag structures with concave surfaces (FACS) electrodeposited on ITO glass (nanoparticles well-separated)	Immersion in analyte solution for 2.5 hrs, followed by drying with N_2	R6G	10^{-12} M R6G (dye)	≈15% RSD (uniformity)	Immersion in KNO_3 or ethanol solution for 44 hrs	44 hrs	2	n.r.	R6G, adenine, 4-ATP	no	
Rinsing	34	Au nanoplates modified with lucigenin	Immersion in analyte solution, followed by drying	pyrene (polycyclic aromatic hydrocarbon pollutant)	10^{-10} M	n.r.	Rinsing with methanol (time not specified)	n.r.	4	≈ 13% RSD*	pyrene (polycyclic aromatic hydrocarbon pollutant)	no	
Rinsing and sonication	35	Au nanostars grafted onto glass and coated with a 1-nm layer of silica	Sample solution drop-cast onto SERS substrate and covered with glass coverslip to form thin film of solution	R6G	0.5 x 10^{-7} M R6G (dye)	n.r.	Rinsing with DI, followed by ultrasonication for 1 min	At least 1 min	n.r.	n.r.	R6G	no	"Many cycles" possible (exact number unspecified) but only data from 1 cycle demonstrated
Rinsing under flow	36	Electrochemically roughened brass plate with electrochemically coated Ag	Sample solution injected in flow-injection analysis system	Nicotinic acid and pyridine	1.7 mM	n.r.	Rinsing for 1 minute with analyte solvent, followed by rinsing with rinsing solution (3 M KCl or 0.1 M NaOH) for 2 minutes	At least 3 min	30	≈85% decrease* from cycle 1 to 30; from cycle 11-30, 5.1% RSD	Nicotinic acid and pyridine	yes	Significant loss of SERS activity after first cleaning cycle; no more than 20 cycles recommended due to reduced performance; low sensitivity
Rinsing under flow	37	Ag thermally deposited on anodized aluminium oxide	Small sample volume (100 nL) injected in flow cell through small capillary. Hydrodynamic focusing of sample stream achieved by pumping sheath water into flow chamber	R6G	10^{-9} M R6G (dye)	n.r.	Sample capillary is flushed with rinsing solution (0.1 M NaOH) under sheath flow	At least 30 s	2	n.r.	R6G	yes	
Rinsing under flow	38	AgNPs embedded in a 3D hydrogel matrix; integrated into a PDMS microfluidic flow cell	Sample injected into microfluidic flow cell and through hydrogel matrix for in situ SERS detection	γ-hydroxybutric acid (GHB) in alcohol	0.08 M GHB<br>0.1 x 10^{-6} M R6G (dye)	6.46% CV reproducibility<br>4.6% CV reliability	5 minutes rinsing with methanol flowed under pressure through hydrogel matrix	5 min	20	≈ 1.3% RSD*	R6G, GHB, o-, m-, and p-aminobenzoic acid	yes	Analyte switching also demonstrated
Works that report removal of adsorbates via competitive binding												
Competitive binding for removing contaminants	39	Electrochemically roughened Ag foil	n/a	n/a	n/a	n/a	SERS substrate is placed in 0.010 M dodecanethiol in methanol solution for 20-24 hours to form SAM and displace any surface contaminants. Afterwards, substrate is treated with ozone, rinsed with methanol for 2 minutes and dried with N_2	At least 20 hours	n/a	n/a	carbonaceous surface contamination; probe molecule: trans-1,2-bis(4-pyridyl)ethylene (BPE)	no	Removal of carbonaceous surface contamination leads to improved background signal
Competitive binding for removing contaminants	40	AuNPs bound onto ITO electrode with APTMS (NPs well-separated)	n/a	n/a	n/a	n/a	Immersed in 1 mM KI for 10 minutes, followed by washing with DI 3x	At least 10 min	n/a	n/a	Surface impurities	no	Surface Au-I bond is strong, so only analytes with strong affinity to gold can be detected, as they need to

													displace the iodide from the iodide-modified surface.
Competitive binding with hydride	41	AuNPs, aggregated with KCl for SERS analysis	n/a	n/a	n/a	n/a	Incubation of AuNPs with >25 mM $NaBH_4$ for 10 minutes for complete desorption of organothiols; other adsorbates may take shorter time for desorption	At least 10 min	2	n.r.	model organothiols: p-methylbenzenethiol (p-MBT), 2-naphthalenethiol (2-NT), p-benzenedithiol (p- BDT), and homocysteine (Hcy); thiophene, adenine, rhodamine, small anions (Br^- and I^-), and a polymer (PVP, poly(N-vinylpyrrolidone))	n/a	
Competitive binding with hydride	42	AuNPs self-assembled on a glass capillary (via APTMS attachment) and coated with SiO_2 layer (Au@SiO_2)	Sample flowed through Au@SiO_2-coated glass capillary and retained there for 30 min, followed by rinsing with DI and ethanol	Thiophenol and malachite green	5x10^{-7} M thiophenol	10-20%	Flow 5.0 x 10^{-2} M of $NaBH_4$ solution through glass capillary for 30 s	20 s	10	≈15 % RSD*	100 µM thiophenol, malachite green; 1,4-benzenedithiol, 4-nitrothiophenol, 4-MBA	yes	Switching also demonstrated with different analytes
Competitive binding with hydride	43	AuNPs/polyvinyl chloride (PVC) film	Immersion in analyte solution for 30 min, followed by rinsing with EtOH and drying in air	Thiram	10 ng cm^{-2}	10.24% RSD (uniformity) 12.54% (batch-to-batch)	$NaBH_4$ solution washing	n.r.	7	≈ 7% RSD*	Thiram	no	
Competitive binding with hydride	44	bimetallic Au/Pt periodical gold gratings	SERS substrate immersed in analyte solution for 20 minutes, washed by ethanol, and dried in a desiccator	dibenzothiophene (fuel impurity)	10^{-12} M	9.1% RSD	5 mL cyclohexene added to SERS substrate, followed by illumination with LED light (850 nm, 200 mW) for 15 minutes to generate active hydrogen *in situ*, then washing with ethanol and acetone	At least 15 min	4	decrease in SERS signal by 4.8% from cycle 1 to 4; increase in spatial variance noted	dibenzothiophene (fuel impurity)	no	Other solvents were tried as active hydrogen sources (water and methanol) but were ineffective in removing the hydrophobic analyte from Pt surface.
Works that report use of electrochemical strategies for SERS substrate recycling													
Electrostatic Adsorption/ Desorption	45	Au-capped Si nanopillars	SERS substrate pre-conditioned 3x (1 min at -0.8V vs Ag/AgCl, and 1 min 0.8V in PBS) to ensure wettability of substrate; SERS substrate then immersed in analyte solution at -0.8V for 30 s prior to SERS mapping (3 min and 30s).	melamine in PBS or milk, pH 3.6 (positively charged)	0.01 ppm in PBS 0.3 ppm in milk	30% RSD between chips	bound analyte removed by applying a positive potential (+0.80 V) for 1 minute after SERS measurements.	At least 1 min	10	11.4-23.7% RSD; ≈40% decrease* in signal from cycle 1 to 10	melamine in PBS or milk, pH 3.6 (positively charged)	yes	60-80 cycles used for calibration curve
Electrochemical reduction (initial cleaning)	46	Roughened Ag electrode (ORCs in 0.1 M KCl)	Immersion in 10 mM thiol solution for 3-24 hrs	n/a	n/a	n/a	Carbonaceous impurities removed by applying negative potential -1.1 V, -1.5 V, or -1.8 V for 120s in 0.1 M NaF	At least 120 s	n/a	n/a	Carbonaceous contamination	no	Negative potential selectively removes carbonaceous impurities. Re-immersion in thiol solution ensures formation of complete SAM
Electrochemical reduction, flow	47–50	*In situ* roughened Ag electrode integrated into electrochemical flow cell	Initial Ag surface conditioning (oxidation-reduction cycle – ORC) +0.4V for 20 s, then back to -0.6V before injection of analyte into flow cell. Analyte signal enhanced at -0.6V	Pyridine, nucleotide bases (adenine, thymine, cytosine, adenosine, adenosine 3-monophosphate	≈ 10^{-6} M	n.r.	Analyte desorbed at -1.2 to -2.0 V vs SCE for 30 s. SERS substrate surface renewed with *in situ* 1s pulse at +0.40 V with analyte present in the cell	10-60 s	2-4	3-7% RSD	Pyridine, adenine, cytosine, adenosine, adenosine 3-monophosphate	yes	"*In situ* roughening": Ag electrode roughened in the presence of analyte solution. More negative potentials needed to remove nucleotide bases compared to pyridine

Electrochemical reduction, flow	51	Polycrystalline Ag electrode roughened *ex situ* integrated into electrochemical flow cell	Incubation of SERS substrate in analyte solution, followed by enhancement of analyte adsorption at -1.2 V vs SCE	Melamine at pH 7 (neutral)	5 x 10^{-8} M melamine in 0.1 M KCl	n.r.	Analyte desorbed at -2.0 V vs SCE for 20 s in alkaline solution (NaOH, Na_2CO_3 and Na_3PO_4). SERS substrate surface re-roughened by applying +0.40 V vs SCE until 130 mC cm^{-2} charge is passed	At least 20 s	10	15% RSD	Melamine at pH 7 (neutral)	yes	"*Ex situ* roughening": Ag electrode roughened in electrolyte only (no analyte present)
Electrochemical reduction (initial cleaning)	40	AuNPs bound onto ITO electrode with APTMS (NPs well-separated)	n/a	n/a	n/a	n/a	Impurities removed by holding SERS substrate at -0.7V for 10 mins in 0.1 M $NaClO_4$. Potential held while rinsing with fresh $NaClO_4$, then rinsed with DI	At least 10 min	n/a	n/a	Surface impurities	no	
Electrochemical, flow	52	Inkjet-printed AgNP spots on ITO integrated into microfluidic flow cell with Pt counter electrode	Analyte solution injected into microfluidic cell with flow rate of 5 µL min^{-1}	n/a	n/a	n/a	Simultaneous flow of phosphate buffer (pH 3, 22.89 mM at 5 µL min^{-1} flow rate) and application of 100 V for 300 s	At least 300 s	3	33-40% RSD	brilliant green, malachite green, crystal violet	yes	Electrochemical system is unreferenced, so mechanism of cleaning is difficult to elucidate. Required potentials and times noted to vary per substate
Electrochemical, flow	53	Porous Ag wire integrated into microfluidic flow cell with Cu counter electrode	Analyte solution injected into microfluidic flow cell	n/a	n/a	n/a	Injection and rinsing with 66 mM phosphate buffer, pH 7 at 50 µL min^{-1} for 10 minutes, followed by application of 4 V for 40 s	At least 40 s	18 cycles with 10 µM CV. Switch between MG and CV also demonstrated for 4 cycles	≈18% RSD* ≈30% decrease* in analyte signal from cycle 1 to 18	malachite green (MG), crystal violet (CV)	yes	
Competitive binding with electrochemical desorption (*initial clean*)	54	AuNPs deposited on APTMS-functionalized ITO	n/a	n/a	n/a	n/a	Au/ITO substrates immersed in 1 mM KI solution for 10 min; followed by electrochemical oxidation ≈0.8V-1.0V in 0.1 M $NaClO_4$	At least 10 min	1	50% decrease in SERS activity	10 mM pyridine, surface impurities	no	Iodide ion displaces surface impurities. I^- then removed electro-chemically by oxidizing it to IO_3^-. Decrease in SERS activity due to dissolution of Au with adsorbed I^-
Competitive binding with electrochemical desorption	51	Polycrystalline Ag electrode roughened ex situ with oxidation-reduction cycles (ORC)	Incubation of SERS substrate in analyte solution, followed by electrochemical enhancement of analyte adsorption at -1.2 V vs SCE	Melamine at pH 7 (neutral)	5 x 10^{-8} M melamine in 0.1 M KCl	n.r.	SERS substrate with adsorbed analyte is incubated for 40 s in 10^{-3} M cetylpyridium chloride (CPC) effectively displacing analyte. To remove adsorbed CPC, the potential is stepped to -1.3 V vs SCE	At least 40 s	10	10% RSD	Melamine at pH 7 (neutral)	yes	
Electrochemical etching and redeposition	55	Glassy carbon electrode with Ag electrodeposited *in situ*. Electrode integrated into electrochemical flow cell	Analyte solution flown through electrochemical flow cell at 1 mL min^{-1} at -1.3 V	Fe(II) in the form of $[Fe(bipy)_3]^{2+}$	10^{-9} M	10% RSD	Ag film anodically stripped by scanning potential from -1.3 V to +0.8V at 20 mV s^{-1}. New Ag film deposited by injecting 400 µL 0.050 M K_2SO_4 and 7.2 x 10^{-4} M $AgNO_3$ and scanning back to -1 V at 20 mV s^{-1}	At least 195 s	n.r.	n.r.	Fe(II) in the form of $[Fe(bipy)_3]^{2+}$	yes	$[Fe(bipy)_3]^{2+}$ cannot be removed with negative or positive potentials
Electrochemical etching and redeposition	56	Ag electrodeposited on Au@SiO_2	Analyte solution drop-cast onto substrate, incubated for 1 hr, rinsed with water, then dried under vacuum	RhB	10^{-11} M RhB (dye)	n.r.	Ag shell and analytes stripped by holding potential at 0.6 V vs. SCE in 0.1 M H2SO4 for 10 min. Ag reelectrodeposited at -0.4 V vs SCE in 0.1 M AgNO3 for 1 min	At least 11 min	10	10% decrease in SERS activity from cycle 1 to 10	RhB, NADPH	no	
Electrochemical etching and redeposition	57	AgNP-coated Au nanorod vertical array (AgNP/GNR) on ITO electrode	Incubation in analyte solution (time not reported), followed by evaporation of solvent	4-ATP, amoxicillin, tetracycline, ofloxacin	0.25 x 10^{-9} M 4-ATP	7.44% RSD (uniformity)	Analytes and Ag shell are stripped off by applying -0.80 V (vs SCE) in 20 mM H_2SO_4 for 120 s. Fresh Ag is then electrodeposited on the AuNR array through a potential	At least 4 minutes	10	≈ 6% RSD* 12% decrease in SERS activity from cycle 1 to 10	4-ATP, amoxicillin, tetracycline, ofloxacin	no	

							step of -0.20 V vs. SCE in 20 mM $AgNO_3$ for 120 s.						
Electrochemical oxidation and reduction	This work	Thin-film AuNP aggregate with CB[5] scaffolds, deposited on FTO-coated glass	Analyte solution pumped through electrochemical flow cell. -0.60 V enhancement potential applied for 15 s	Adenine (ADN)	2 x 10^{-7} M ADN in buffer	4-5.7% RSD (regional uniformity)	(1) +1.5 V applied for 10-60 s in 50 mM potassium phosphate buffer (pH 7.0). (2) -0.80 V applied for 5 s in 1 mM CB[5] in 50 mM buffer	At least 15s	30	1.0-6.7% RSD	Adenine, cytosine, thiols, hypoxanthine, creatinine, nicotinamide, paracetamol, norepinephrine, tryptophan, nicotinic acid, and methylene blue	yes	

Supplementary References

1. Arul, R. *et al.* Giant mid-IR resonant coupling to molecular vibrations in sub-nm gaps of plasmonic multilayer metafilms. *Light Sci. Appl.* **11**, 281 (2022).
2. Mahajan, S. *et al.* Raman and SERS spectroscopy of cucurbit[n]urils. *Phys. Chem. Chem. Phys.* **12**, 10429–10433 (2010).
3. Pfisterer, J. H. K. *et al.* Role of OH Intermediates during the Au Oxide Electro-Reduction at Low pH Elucidated by Electrochemical Surface-Enhanced Raman Spectroscopy and Implicit Solvent Density Functional Theory. *ACS Catal.* **10**, 12716–12726 (2020).
4. Zhumaev, U. *et al.* Electro-oxidation of Au(1 1 1) in contact with aqueous electrolytes: New insight from in situ vibration spectroscopy. *Electrochim. Acta* **112**, 853–863 (2013).
5. Niaura, G., Gaigalas, A. K. & Vilker, V. L. Surface-enhanced Raman spectroscopy of phosphate anions: Adsorption on silver, gold, and copper electrodes. *J. Phys. Chem. B* **101**, 9250–9262 (1997).
6. Gao, P. & Weaver, M. J. Metal-adsorbate vibrational frequencies as a probe of surface bonding: Halides and pseudohalides at gold electrodes. *J. Phys. Chem.* **90**, 4057–4063 (1986).
7. Loo, B. H. In situ identification of halide complexes on gold electrode by surface-enhanced Raman spectroscopy. *J. Phys. Chem.* **86**, 433–437 (1982).
8. Juodkazis, K., Juodkazytė, J., Juodienė, T. & Lukinskas, A. Determination of Au(III) in the surface layers formed anodically on the gold electrode. *J. Electroanal. Chem.* **441**, 19–24 (1998).
9. Stumm, C. *et al.* Reduction of Oxide Layers on Au(111): The Interplay between Reduction Rate, Dissolution, and Restructuring. *J. Phys. Chem. C* **125**, 22698–22704 (2021).
10. Van Duyne, R. P. *Laser Excitation of Raman Scattering From Adsorbed Molecules on Electrode Surfaces*. *Chemical and Biochemical Applications of Lasers* (Academic Press, Inc., 1979). doi:10.1016/b978-0-12-505404-1.50009-x.
11. Eliaz, N. & Gileadi, E. *Physical Electrochemistry: Fundamentals, Techniques, and Applications*. (2019).
12. Gopalakrishnan, A. *et al.* Bimetallic 3D nanostar dimers in ring cavities: Recyclable and robust surface-enhanced Raman scattering substrates for signal detection from few molecules. *ACS Nano* **8**, 7986–7994 (2014).
13. Farrell, M. E., Strobbia, P., Pellegrino, P. M. & Cullum, B. Surface regeneration and signal increase in surface-enhanced Raman scattering substrates. *Appl. Opt.* **56**, B198 (2017).
14. Wu, K. *et al.* Gold Nanoparticles Sliding on Recyclable Nanohoodoos—Engineered for Surface-Enhanced Raman Spectroscopy. *Adv. Funct. Mater.* **28**, (2018).
15. Mahurin, S. M., John, J., Sepaniak, M. J. & Dai, S. A reusable surface-enhanced Raman scattering (SERS) substrate prepared by atomic layer deposition of alumina on a multi-layer gold and silver film. *Appl. Spectrosc.* **65**, 417–422 (2011).
16. Cai, Q. *et al.* Boron nitride nanosheets as improved and reusable substrates for gold nanoparticles enabled surface enhanced Raman spectroscopy. *Phys. Chem. Chem. Phys.* **17**, 7761–7766 (2015).
17. Zhang, X. *et al.* Improved Thermal Stability of Graphene-Veiled Noble Metal Nanoarrays as Recyclable SERS Substrates. *ACS Appl. Mater. Interfaces* **9**, 40726–40733 (2017).
18. Liu, D. *et al.* Detection and plasma assisted degradation of dye on reusable gold coated tungsten nanofuzz array surface-enhanced Raman scattering substrate. *Appl. Surf. Sci.* **469**, 262–268 (2019).
19. Negri, P., Marotta, N. E., Bottomley, L. A. & Dluhy, R. A. Removal of surface contamination and self-assembled monolayers (SAMs) from silver (Ag) nanorod substrates by plasma cleaning with argon. *Appl. Spectrosc.* **65**, 66–74 (2011).
20. Song, J., Nam, W. & Zhou, W. Scalable High-Performance Nanolaminated SERS Substrates Based on Multistack Vertically Oriented Plasmonic Nanogaps. *Adv. Mater. Technol.* **4**, 1–7 (2019).
21. Shvalya, V. *et al.* Reusable Au/Pd-coated chestnut-like copper oxide SERS substrates with ultra-fast self-recovery. *Appl. Surf. Sci.* **517**, 146205 (2020).
22. Yang, X., Zhong, H., Zhu, Y., Shen, J. & Li, C. Ultrasensitive and recyclable SERS substrate based on Au-decorated Si nanowire arrays. *Dalt. Trans.* **42**, 14324–14330 (2013).
23. Zou, B., Niu, C., Ma, M., Zhao, L. & Wang, Y. Magnetic Assembly Route to Construct Reproducible and Recyclable SERS Substrate. *Nanoscale Res. Lett.* **14**, (2019).
24. Kang, H.-S. *et al.* High-index facets and multidimensional hotspots in Au-decorated 24-faceted PbS for ultrasensitive and recyclable SERS substrates. *J. Mater. Chem. C* **10**, 958–968 (2022).
25. Yang, Q. *et al.* Hierarchically rough CuO/Ag composite film with controlled morphology as recyclable SERS-active substrate. *Appl. Surf. Sci.* **598**, 153746 (2022).
26. Korcoban, D. *et al.* Recyclable SERS substrate: Optimised by reducing masking effect through colloidal lithography. *Appl. Surf. Sci.* **578**, 151852 (2022).
27. Li, X. *et al.* Ordered array of gold semishells on TiO 2 spheres: An ultrasensitive and recyclable SERS substrate. *ACS Appl. Mater. Interfaces* **4**, 2180–2185 (2012).
28. Shondo, J. *et al.* Nanoscale Synergetic Effects on Ag–TiO 2 Hybrid Substrate for Photoinduced Enhanced Raman Spectroscopy (PIERS) with Ultra-Sensitivity and Reusability. *Small* **18**, 2203861 (2022).
29. Álvarez-Puebla, R. A., Contreras-Cáceres, R., Pastoriza-Santos, I., Pérez-Juste, J. & Liz-Marzán, L. M. Au@pNIPAM colloids as molecular traps for surface-enhanced, spectroscopic, ultra-sensitive analysis. *Angew. Chemie - Int. Ed.* **48**, 138–143 (2009).
30. Zheng, Y. *et al.* Reversible gating of smart plasmonic molecular traps using thermoresponsive polymers for single-molecule detection. *Nat. Commun.* **6**, 1–8 (2015).
31. Guselnikova, O. *et al.* Large-scale, ultrasensitive, highly reproducible and reusable smart sers platform based on pnipam-grafted gold grating. *ChemNanoMat* **3**, 135–144 (2017).

32. Plou, J., Charconnet, M., García, I., Calvo, J. & Liz-Marzán, L. M. Preventing Memory Effects in Surface-Enhanced Raman Scattering Substrates by Polymer Coating and Laser-Activated Deprotection. *ACS Nano* **15**, 8984–8995 (2021).
33. Bian, J. *et al.* Reproducible and recyclable SERS substrates: Flower-like Ag structures with concave surfaces formed by electrodeposition. *Appl. Surf. Sci.* **333**, 126–133 (2015).
34. Lin, W. H., Lu, Y. H. & Hsu, Y. J. Au nanoplates as robust, recyclable SERS substrates for ultrasensitive chemical sensing. *J. Colloid Interface Sci.* **418**, 87–94 (2014).
35. Bassi, B. *et al.* Robust, reproducible, recyclable SERS substrates: Monolayers of gold nanostars grafted on glass and coated with a thin silica layer. *Nanotechnology* **30**, (2019).
36. Weißenbacher, N., Lendl, B., Frank, J., Wanzenböck, H. D. & Kellner, R. Surface enhanced Raman spectroscopy as a molecular specific detection system in aqueous flow-through systems. *Analyst* **123**, 1057–1060 (1998).
37. Negri, P., Jacobs, K. T., Dada, O. O. & Schultz, Z. D. Ultrasensitive Surface-Enhanced Raman Scattering Flow Detector Using Hydrodynamic Focusing. *Anal. Chem.* **85**, 10159–10166 (2013).
38. Shin, Y. *et al.* Facile Microfluidic Fabrication of 3D Hydrogel SERS Substrate with High Reusability and Reproducibility via Programmable Maskless Flow Microlithography. *Adv. Opt. Mater.* **8**, 1–10 (2020).
39. Norrod, K. L. & Rowlen, K. L. Removal of Carbonaceous Contamination from SERS-Active Silver by Self-Assembly of Decanethiol. *Anal. Chem.* **70**, 4218–4221 (1998).
40. Huang, J. Y., Zong, C., Xu, L. J., Cui, Y. & Ren, B. Clean and modified substrates for direct detection of living cells by surface-enhanced raman spectroscopy. *Chem. Commun.* **47**, 5738–5740 (2011).
41. Ansar, S. M. *et al.* Removal of Molecular Adsorbates on Gold Nanoparticles Using Sodium Borohydride in Water. *Nano Lett.* **13**, 1226–1229 (2013).
42. Wang, W. *et al.* On-line surface enhanced Raman spectroscopic detection in a recyclable Au@SiO2 modified glass capillary. *J. Raman Spectrosc.* **45**, 736–744 (2014).
43. Wu, P., Zhong, L. Bin, Liu, Q., Zhou, X. & Zheng, Y. M. Polymer induced one-step interfacial self-assembly method for the fabrication of flexible, robust and free-standing SERS substrates for rapid on-site detection of pesticide residues. *Nanoscale* **11**, 12829–12836 (2019).
44. Guselnikova, O. *et al.* Plasmon-assisted self-cleaning sensor for the detection of organosulfur compounds in fuels. *J. Mater. Chem. C* **7**, 14181–14187 (2019).
45. Viehrig, M. *et al.* Quantitative SERS Assay on a Single Chip Enabled by Electrochemically Assisted Regeneration: A Method for Detection of Melamine in Milk. *Anal. Chem.* **92**, 4317–4325 (2020).
46. Schoenfisch, M. H., Ross, A. M. & Pemberton, J. E. Electrochemical cleaning of surface-confined carbon contamination in self-assembled monolayers on polycrystalline Ag and Au. *Langmuir* **16**, 2907–2914 (2000).
47. Forcé, R. K. Surface-Enhanced Raman Spectroscopy at a Silver Electrode as a Detector in Flow Injection Analysis. *Anal. Chem.* **60**, 1987–1989 (1988).
48. Pothier, N. J. & Force, R. K. Surface-enhanced Raman spectroscopy at a silver electrode as a detection system in flowing streams. *Anal. Chem.* **62**, 678–680 (1990).
49. Pothier, N. J. & Forcé, R. K. Surface-Enhanced Raman Spectroscopy at a Silver Electrode as a Real-Time Detector in Flowing Streams. *Appl. Spectrosc.* **46**, 147–151 (1992).
50. Pothier, N. J. & Force, R. K. Detection of biologically important compounds in flowing aqueous streams by surface-enhanced Raman spectroscopy at a silver electrode. *Appl. Spectrosc.* **48**, 421–425 (1994).
51. Koglin, E., Laumen, B., Borgarello, E. & Borgarello, G. Competitive and displacement adsorption of cetylpyridinium chloride and p-nitrophenol on charged surfaces: A surface-enhanced Raman microprobe Scattering (SERS) study. *Prog. Colloid Polym. Sci.* **95**, 143–152 (1994).
52. Meier, T. A. *et al.* Fast electrically assisted regeneration of on-chip SERS substrates. *Lab Chip* **15**, 2923–2927 (2015).
53. Höhn, E.-M., Panneerselvam, R., Das, A. & Belder, D. Raman Spectroscopic Detection in Continuous Microflow Using a Chip-Integrated Silver Electrode as an Electrically Regenerable Surface-Enhanced Raman Spectroscopy Substrate. *Anal. Chem.* **91**, 9844–9851 (2019).
54. Li, M. De *et al.* Clean substrates prepared by chemical adsorption of iodide followed by electrochemical oxidation for surface-enhanced raman spectroscopic study of cell membrane. *Anal. Chem.* **80**, 5118–5125 (2008).
55. Gouveia, V. J. P., Gutz, I. G. & Rubim, J. C. A new spectroelectrochemical cell for flow injection analysis and its application to the determination of Fe(II) down to the femtomol level by surface-enhanced resonance Raman scattering (SERRS). *J. Electroanal. Chem.* **371**, 37–42 (1994).
56. Li, D., Li, D.-W., Li, Y., Fossey, J. S. & Long, Y.-T. Cyclic electroplating and stripping of silver on Au@SiO2 core/shell nanoparticles for sensitive and recyclable substrate of surface-enhanced Raman scattering. *J. Mater. Chem.* **20**, 3688 (2010).
57. Peng, X., Li, D., Li, Y., Xing, H. & Deng, W. Plasmonic tunable Ag-coated gold nanorod arrays as reusable SERS substrates for multiplexed antibiotics detection. *J. Mater. Chem. B* **9**, 1123–1130 (2021).
